# Role of Mineral Surfaces in Prebiotic Chemical Evolution. In Silico Quantum Mechanical Studies

**DOI:** 10.3390/life9010010

**Published:** 2019-01-17

**Authors:** Albert Rimola, Mariona Sodupe, Piero Ugliengo

**Affiliations:** 1 Departament de Química, Universitat Autònoma de Barcelona, 08193 Bellaterra, Spain; mariona.sodupe@uab.cat; 2 Dipartimento di Chimica and Nanostructured Interfaces and Surfaces (NIS), Università degli Studi di Torino, Via P. Giuria 7, 10125 Torino, Italy

**Keywords:** prebiotic chemistry, theoretical chemistry, surface modelling, mineral surfaces, early earth, density functional theory, origin of life

## Abstract

There is a consensus that the interaction of organic molecules with the surfaces of naturally-occurring minerals might have played a crucial role in chemical evolution and complexification in a prebiotic era. The hurdle of an overly diluted primordial soup occurring in the free ocean may have been overcome by the adsorption and concentration of relevant molecules on the surface of abundant minerals at the sea shore. Specific organic–mineral interactions could, at the same time, organize adsorbed molecules in well-defined orientations and activate them toward chemical reactions, bringing to an increase in chemical complexity. As experimental approaches cannot easily provide details at atomic resolution, the role of in silico computer simulations may fill that gap by providing structures and reactive energy profiles at the organic–mineral interface regions. Accordingly, numerous computational studies devoted to prebiotic chemical evolution induced by organic–mineral interactions have been proposed. The present article aims at reviewing recent in silico works, mainly focusing on prebiotic processes occurring on the mineral surfaces of clays, iron sulfides, titanium dioxide, and silica and silicates simulated through quantum mechanical methods based on the density functional theory (DFT). The DFT is the most accurate way in which chemists may address the behavior of the molecular world through large models mimicking chemical complexity. A perspective on possible future scenarios of research using in silico techniques is finally proposed.

## 1. Introduction

With respect to the period ranging from the Big Bang (~14.5 Ga) to the emergence (and survival) of the first forms of life (estimated at ~3.7 Ga), there is a consensus that the process took place through a set of organizational events. Generally speaking, physics provide models to explain the Big Bang, the formation of subatomic particles, and the formation of H and He and heavy atoms in space. Chemistry comes in when one aims to understand how atoms first combine to form molecules (i.e., covalent bonds at work), and how they interact to form supramolecular systems (i.e., non-covalent interactions at work). The study of the emergence of the first self-replicating systems, their perpetuation, and further evolution into modern biochemical systems forms the realm of biology.

The critical step in which a non-replicator (supramolecular) system converts into a self-replicating (biochemical) system is still not known and is open to investigation. In this context, the debate between “genetics first” or “metabolism first” is still in play [1,2,3,4,5,6], although the models are not mutually exclusive. The first paradigm advocates that RNA (or a primitive form of it) emerged as the first system capable of self-replicating, which in turn could perform enzymatic processes. The second paradigm supports that metabolic processes assembled prior to the existence of replicators, with these emerging as products of the increasing activity of the proto-metabolic cycles.

Systems chemistry (SC), also referred to as adaptive chemistry, is one of the current research lines focused on understanding the “supramolecular → self-replication” transition [7,8,9,10,11,12]. This sub-discipline of chemistry does not address the properties of individual chemical components but the properties of a collective (ensemble) of molecular components. These ensembles consist of an overall network of interacting molecules at different hierarchical levels, which present different properties compared with the individual components. The underlying idea of SC is that, at variance with a traditional chemical system which lives at the bottom of the Gibbs free energy landscape and, accordingly, maps the most stable states, SC ensembles are not necessarily thermodynamically stable. In fact, they are in a state that is far from equilibrium and, most important, dissipative. This means that molecules belonging to a SC ensemble continuously consume energy to perform specific functions, which can be different with respect to their isolated state. Therefore, the SC ensembles can exhibit unexpected and emergent properties, such as complex reaction networks that can eventually led to jump-starting the emergence of self-replicating systems.

Let us now focus on prebiotic chemical evolution (CE). CE envisages matter evolving from simple forms to systems of increasing complexity. In a prebiotic context, the CE sequence starts from atoms, passing through the molecular chemistry up to reach supramolecular chemistry. Self-organization is a fundamental aspect in CE, and mineral surfaces could have played an essential role in this process by selecting and concentrating compounds and templating and finally catalysing reactions of prebiotic interest [13]. Plausible scenarios of the primordial prebiotic CE involve processes occurring either at the surface of interstellar dust grains or at the surfaces of the crust in the Hadean Eon of Earth. Minerals were ubiquitously present in both, presenting potential opportunities for concentration of the raw material and providing surfaces able to act as heterogeneous catalysts.

In space, most of the atoms formed via nucleosynthesis combine to form molecules. Circa 200 gas-phase molecular species have been detected by means of rotational spectroscopic observations [14,15,16,17,18]. Among the molecules detected, one can find essential molecules (e.g., H_2_, CO, H_2_O, or NH_3_), the so-called complex organic molecules (COMs, which are organic compounds between 6 and 13 atoms (e.g., CH_3_OH, NH_2_CHO, or CH_3_CHO)), and also large carbonaceous compounds such as linear C-chains (e.g., HC_5_N or HC_7_O) and fullerene derivatives (e.g., C_60_ or C_70_). A classification list of these identified compounds [19] perfectly reflects the chemical evolution (and diversity) occurring in space. This molecular complexity seems to go hand-in-hand with the different phases of the formation of a star and a planetary system [20], since more complex molecules form at each step. Most of these molecules are synthesized via gas-phase processes, but some reactions require the presence of solid grains providing the surfaces where to occur. These grains consist of silicates [21], carbonaceous materials [22], and dirty ices (ices of water with other minor amounts of volatile species such as CO or NH_3_) [23]. Two paradigmatic cases are H_2_ [24] and H_2_O [25], the most abundant molecules in space in the gas phase and the solid phase, respectively. Their formation in pure gas-phase conditions is very inefficient, while on cosmic grains the reactions are largely favoured. Other essential species synthesized by surface reactions are NH_3_, CH_4_, CO_2_, formaldehyde, and methanol, just to mention few of the available COMs [26]. Moreover, comets and meteorites (particularly those classified as carbonaceous chondrites) present a significant (up to 5%) content of organic matter, including that of biological relevance such as amino acids, nucleobases and sugars [27,28,29,30]. The solid components of these asteroidal bodies (i.e., a rich variety of minerals) could have acted as active surface catalysts for the synthesis of these complex molecules. This was demonstrated by Earth Laboratories for reactions undergone by formamide [31].

The presumable anoxia of Earth’s Hadean Eon atmosphere was due to its composition of mainly volcanic gases [32] (i.e., H_2_O, CO_2_, SO_2_, H_2_S, and N_2_) with small amounts of CO and H_2_, liquid water, and mineral rocks. The experiments of Miller–Urey [33,34] and Oró [35] supposed a turning point in the understanding of the prebiotic chemical evolution, since complex organic compounds were synthesized from simpler inorganic precursors. Because of its rocky nature, the early Earth’s crust consisted of naturally-occurring minerals, the surfaces of which provided catalytic sites that could contribute to a prebiotic organic complexification. Recently, Hazen (see Tables 1 and 2 of [36]) has undertaken a deep analysis of the potentially present minerals in the Hadean Eon, encompassing Earth’s first 550 million years, reaching the conclusion that about 420 out of the total 4800 current minerals were present. The selection was based on the constraint that they should be widely distributed and/or volumetrically significant as well as compatible with the anoxic character of the early atmosphere. To stress the relevance of minerals in prebiotic chemistry one can focus on formamide, one of the most important COMs. There is indeed experimental evidence that formamide is a potential source of HCN and NH_3_ when it decomposes which, in turn, give rise to several molecular building blocks of biological relevance [37,38,39]. These reactions, however, only take place in the presence of minerals, e.g., silica, alumina, titania, or clays, all present in the Hadean Eon, highlighting the catalytic role of these minerals. The iron–sulphur world theory advocates the role of iron sulphides and their redox properties as crucial components towards chemical complexity [40,41]. The particularity of this theory is that the chemical evolution is achieved by autocatalytic surface metabolism of small organic molecules on the minerals. That is, proto-metabolic cycles occur on the mineral surfaces, these acting as “inorganic enzymes”. On an early Earth, this scenario could have operated in hydrothermal deep-sea vents (black smokers) at high pressure and temperature, where iron sulphide minerals were available abundantly, providing conditions suitable for prebiotic redox processes. Cleverly designed experiments simulating the same physico-chemical conditions at the “black smokers” confirmed the power of hydrothermal organic synthesis [42,43]. This scenario is particularly attractive conceptually, as processes occurring in the deep sea are protected from the sterilizing events occurring at the surface of the young Earth (extra-terrestrial body bombardment). In a more general physico-chemical view, Shock and co-workers [44,45] showed that hydrothermal circulation at seafloor brings fluid mixing of water with a source of heat. As aqueous fluids with differing compositions (and oxidation states) are usually far from thermodynamic equilibrium when they mix, they provide a source of free energy driving organic synthesis from CO_2_ and H_2_. This approach culminated with the concept of “geobiochemistry” [46], in which the emergence of life on Earth occurred where catalysis can take place, like in ion-rich hydrothermal vents, to expedite the release of chemical energy in water–rock–organic systems. Within this concept, however, the specific role of mineral surfaces is less essential, the key being instead the dissolved ions coming from either ultramafic or basaltic rocks.

Condensation reactions, i.e., reactions giving rise to water as product, are considered prebiotic processes of high relevance, as they could lead to the formation of the first biopolymers on a primitive Earth. Peptide bond formation (the joining of amino acids to form peptides) or phosphodiester bond formation (the joining of two nucleotides to form a nucleotide strand) are examples of prebiotic condensations. These reactions are thermodynamically disfavoured in liquid water as they release one water molecule for each condensation step. Thus, we are faced with the water paradox [47]: water is a mandatory pre-requisite for life [48] but its presence hampers condensation reactions that are crucial for the emergence of life. Furthermore, the probability of encounter between molecules in a 3D ocean is such that they can never meet. However, as shown in 1921 by Pólya’s theorem [49], on a 2D lattice (like a mineral surface) the probability of an encounter is 1 in a finite time, i.e., adsorbed molecules travelling by small jumps from one adsorbed site to the next-neighbour will finally meet and eventually react. In his seminal work [50], British biophysicist John Desmond Bernal proposed that minerals could have played a key role favouring the reaction, as they present specific surface sites that can adsorb and concentrate prebiotic organic compounds. Almost 20 years ago, Orgel proposed the polymerization on the rocks model [51], which states that bio-oligomers can be elongated by repeated condensation cycles on the mineral up to a length in which adsorption is almost irreversible. Additionally, Smith suggested that minerals might have activated biomolecules for catalytic assembly into specific biopolymers and protected them from prompt hydrolysis and photochemical destruction [52]. The validity of these theories has been widely demonstrated in different experiments, such as those related to the condensation of amino acids in the presence of silica [53,54,55,56,57,58], clays [59,60], alumina [61,62,63,64], iron oxides [65], hydroxides [66], and titania [57,67,68]. In parallel, an interesting hypothesis to overcome the “water paradox” is that based on fluctuating environments in the prebiotic Earth. That is, daily fluctuations of temperature and humidity, which readily occur under natural conditions, could have led to cycles of drying and rewetting, allowing condensation reactions. This theory is supported by experimental evidence [60,69]. Other possible solutions include salt-induced peptide formation as proposed in a study by the group of Rode [70,71,72], in which high concentrations of NaCl and Cu (II) ions (Na^+^ concentrations were above 3 M) acted respectively as dehydrating and complexing agents at temperatures between 60 and 90 degrees Celsius. Other already mentioned approaches (vide supra) are hydrothermal synthesis [73] and the oligomerization of amino acids inside lipid vesicles in a simulated hydrothermal environment in which temperature fluctuation results in heptaglycine in the absence of condensing agents [74]. Peptide synthesis was also shown to be triggered by comet impacts [75,76]. Oligomerization was also proved by coupling a system with glycylglycine to metaphosphate hydrolysis with catalytic support by Mg^2+^ ions [77] or by using diamidophosphate (DAP) derived from trimetaphosphate and (amido)phosphorylates to provide a wide variety of (pre)biological building blocks (nucleosides/tides, amino acids, and lipid precursors) under aqueous conditions, without the need for a condensing agent [78].

As this account focuses on the quantum mechanical simulations of the role of mineral surfaces, the reader interested in a whole account of the details of all possible scenarios compatible with a prebiotic origin of life may refer to [1,2,3,4,5,6,7,8,9], as well more recent work [79].

Therefore, it seems evident that the interaction of mineral surfaces with organic compounds is of great interest for studies involved in the field of prebiotic chemistry. Several experimental techniques to characterize the adsorption of biomolecules on mineral surfaces are available. The least demanding ones from a technical point of view are those that provide macroscopic data, such as adsorption isotherm measurements, which can provide equilibrium constants for adsorption (giving access to the adsorption free energy Δ_ads_G^0^) and saturation coverages. In situ IR measurements during the adsorption process allow for determining vibrational shifts caused by biomolecule–mineral interactions (which can drive fine structural details such as coordination to surface atoms and specific H-bond patterns), and even the appearance of new vibrational features due to product formation upon thermal treatment. Solid state nuclear magnetic resonance (NMR), UV-visible spectroscopy, Raman and X-ray Absorption Spectroscopy (XAS) spectroscopy, and fluorescence methods are also useful techniques. However, getting accurate structural information from an experimental viewpoint is difficult, since only few techniques (e.g., those derived from electronic spectroscopy) can provide atomic resolution information. Therefore, with experiments only, the fine details of the biomolecule/mineral interactions cannot be entirely elucidated due to their complexity. The lack of atomic-scale information can be filled in by means of computer (in silico) modelling and simulation techniques. These methods are becoming, day by day, more essential to obtain both structural atomistic details of the biomolecule/mineral interface regions as well as energetic and vibrational features of the adsorption process, which in turn can be useful to interpret experimental data. Moreover, simulations based on quantum chemical methods can be used to study reactivity by exploring potential energy surfaces associated with a given reaction. With the energy profiles, reaction energies and kinetic constants can be derived, which are useful to assess the plausibility of the reactions in particular conditions.

The present work aims to review the computational chemistry literature exclusively based on quantum mechanical approaches that addressed the interaction of organic compounds with mineral surfaces and their reactivity within the context of prebiotic chemistry. It is worth noting that different reviews on this topic (not only limited to theoretical studies) are already available [80,81,82,83]. Therefore, the current review mainly addresses the new studies that were published afterwards these mentioned reviews, although we also refer to older works which for us are of reference. The review is organized as follows. Section 2 provides a brief description of the quantum chemical methods most often used in prebiotic chemistry studies. Section 3 is devoted to the computational strategies to model mineral surfaces of prebiotic interest. Section 4 is the core of the review, in which the most recent computational works are briefly exposed. Finally, Section 5 consists of the concluding remarks including future perspectives in the in silico mineral-induced prebiotic chemistry field.

## 2. Quantum Mechanical Methods

An accurate description of the prebiotic organic molecule/mineral surface interactions and related chemistry requires the use of quantum mechanical (QM) methods. These methods are based on the resolution of the time-independent electronic Schrödinger equation within the Born–Oppenheimer approximation. It is worth mentioning that in this section we exclude simulations based on molecular mechanics (MMs). These simulations treat molecules as a set of masses and springs of given force constant, which are described by individual potential functions representing bond lengths, angles, torsions, and interatomic interactions, the overall terms constituting a force field (FF). As electrons are not explicitly accounted for, classical FF simulations cannot handle chemical reactions (although FFs capable of describing reactions do exist; e.g., ReaxFF [84]). The most critical aspect of using MMs in surface-induced prebiotic problems is that no reliable FFs have been developed to accurately describe the regions of the organic/mineral interface. Indeed, while specialized FFs for organic and biochemical matter as well as to describe bulk mineral materials exist, unfortunately none of them have been explicitly parametrized to simulate the interaction between these two categories of systems. This task is particularly difficult owing to the fact that chemical reactivity (proton and electron transfer) at the frontier of these systems is normal rather than an exception. Nonetheless, distinguished work at the MM level relevant for the interaction of genetic material with the inner surfaces of clay minerals is provided by Coveney and collaborators [85]. In an elegant study, they assessed the interaction between the 25-mer sequences of single-stranded ribonucleic acid (RNA) in bulk water and at the surface of three hydrated positively-charged MgAl-layered double hydroxide (LDH) minerals [86]. A combination of different force fields was adopted to treat the RNA, LDH, and water in a balanced way. In a similar study [87], the structural stability of three different nucleic acids intercalated within magnesium aluminium-layered double hydroxide mineral, at varying degrees of hydration, and free in aqueous solution was assessed. Using the same MM approach, a recent work has been published on the power of LDH minerals to concentrate, align, and act as adsorption templates for amino acids, and during wetting–drying cycles to promote peptide bond formation [88].

Among QM methods based on the wave function, the simplest one is the Hartree–Fock (HF) method, in which the wave function for an *N*-electron system is described by a single Slater determinant constituted by *N*-one-electron spin orbitals to ensure the minimum electronic energy. The description in terms of independent spin-orbitals and the use of a single determinant cause the neglect of the so-called electron correlation, leading to a poor description of formation/breaking of chemical bond, H-bond, and charge transfer processes. Furthermore, at the Hartree–Fock level, dispersion (London) interactions are also entirely missing, a serious fault when studying the adsorption of relatively large organic molecules with extended mineral surfaces. Accordingly, HF is too approximate for chemically complex problems. Since exact wave functions cannot be described by a single slater determinant, the subsequent wave function based methods (called post-HF) all try to recover the missing electron correlation of the HF method by expanding the wave function. Methods of this kind are those based on the Møller–Plesset perturbation theory (MPn methods), the configuration interaction (CI, in which electron excited configurations are introduced in a very large expansion of the wave-function in terms of Slater determinants), or coupled cluster (CC) methods (in which electron excitations are introduced through an exponential ansatz acting on the HF wavefunction [89]). The CCSD(T) is a CC derivation that extensively includes electron correlation through the (S)ingle, (D)ouble, and (T)triple excitations (albeit the latter in a perturbative way) and, when combined with extended basis sets, is considered the “golden standard” in quantum chemistry [90]. Accordingly, it is usually used to calibrate more approximate methods (e.g., density functional theory, DFT; see below). Post-HF methods may become dramatically expensive for medium/large systems; fortunately, clever coding and optimization (e.g., the “Coupled-Cluster techniques for Computational Chemistry” CFOUR program [91]) have allowed the CCSD(T) evaluation not only of energy but also of analytical gradients and second derivatives for molecules of medium size.

A computationally convenient alternative to post-HF methods is the density functional theory (DFT). This approach is attractive compared to the post-HF ones, because it recovers a significant degree of electron correlation at a computational cost similar to HF. The underlying idea of DFT is that the total ground state energy and related properties of an *N*-electron system can be determined by an universal and exact mathematical functional of the electron density. In DFT, the *N*-electron wave-function, whose complexity grows with the number of particles, is replaced by the electron density, which is a function of only three variables, irrespective on the complexity and size of the system. The development of a broad variety of DFT methods arises from the attempts to build the exact functional. The most recurrent DFT methods are those based on the generalized gradient approximation (GGA) whose most common incarnations are as PBE [92], PW91 [93] or BLYP [94,95], and the hybrid functionals (e.g., B3LYP [95,96], PBE0 [97] or BHLYP [95,98]), which include a fraction of exact exchange of the HF wavefunction in the exchange potential, improving the description of H-bond interactions (overstabilised by GGA) and the electronic structure of systems where the self-interaction error can be important, such as open-shell systems. Newer forms of meta-GGA (e.g., TPSS [99] or M06L [100]) and hybrid meta-GGA (e.g., M06 and M062X [101]), which include kinetic energy density and/or Laplacian terms, have been developed to improve the performances of pure GGA and hybrid functionals. An important drawback of standard DFT methods also shared by HF (vide supra) is that long-range non-covalent (i.e., dispersion-based) interactions are not accounted for in their definition [102]. A pragmatic solution is the DFT-D scheme, in which dispersion contributions are introduced in the form of a posteriori correction (D) on the DFT energies. The D term is based on an atom–atom additive London-type empirical potential scaling as a R^−6^ function of the atom–atom separation R. To avoid double-counting of the interactions, the pure DFT energy is smoothly corrected through a damping function screening out the D contribution at short-range. This DFT-D computationally very cheap scheme was developed by Grimme and co-workers and sequentially improved to obtain accurate dispersion correction terms for any chemical system (D [103], D2 [104], D3 [105] and D4 [106]).

Static calculations are those that do not account for dynamic effects exerted by temperature, i.e., calculations that are performed considering 0 K. Neglecting dynamics effects can have important consequences as they can affect the stability and conformation of the adsorbed species. A usual strategy to include dynamic effects is through the execution of molecular dynamics (MD) simulations, in which the evolution in time–space phase of the atomic positions subject to the internal forces (of chemical nature) and to the kinetic energy due to the temperature of the system can be explored. When molecular dynamics simulations arise from combining electronic structure theory (for electron description) with the classical nuclear motion, the simulations are called ab initio molecular dynamics (AIMD). Standard implementation of a MD simulation is very inefficient when kinetic barriers, as in chemical reactions, have to be sampled. Recently, a methodology known as metadynamics significantly sped up the search for and characterization of reactive free potential energy surfaces. The exploration of complex free potential energy landscapes is then achieved by changing the surface through the dropping of Gaussian functions in local minima during the evolution of the simulation, thus helping the system to explore different configurations [107]. The key problem when running metadynamics, however, is the choice of proper reaction coordinates that bring reactants to products. Path collective variables have been proposed for that purpose [108], but the specific choice remains challenging when complex reactions, like the one occurring for instance in the Urey–Miller experiment, are studied. An interesting and exciting approach has been proposed by Martinez and co-workers, known as the “ab initio nanoreactor” [109]. In essence, AIMD is carried out at the HF level with small basis sets to save computer time in a virtual reactor envisaging a sphere from which molecules cannot escape despite the sudden variations in volume of the sphere, causing, in turn, a steep increase in temperature (up to 10,000 K). To constrain the molecule in order to stay within the sphere, an empirical potential is added to the quantum mechanical Hamiltonian. This mimics the injection of energy in the system through high energy collisions with the consequence that the initial molecular system evolves towards a complex tree of possible products. No a priori choice of the collective variables or alike is established and the system is free to explore the tremendously complex potential energy surface. A machine learning algorithm was then developed to automatically identify new products of the reaction. To focus on the formation path of a particular compound, the local network of closely related compounds (namely, the molecules that appear on either side of chemical equations that lead to the compound of interest) was mapped out. The molecular dynamics pathway that connects stable reactant and product species was used to locate a corresponding minimum-energy path from which activated complexes are defined allowing to arrive at the kinetic energy barriers through a more refined DFT approach. The methodology is fascinating, particularly its technicalities, which are essential for the stability of the simulation. It has been applied to study the reaction product of acetylene reactions and of a virtual Urey–Miller experiment, relevant for prebiotic chemistry. A similar approach has been recently proposed by Saitta and Saija [110] for the in silico simulation of the Urey–Miller experiment, in which the injection of energy is simulated through the effect of intense electric fields across the simulation cell. Simulations were run at zero field as well, to show that no reaction occurred in the starting H_2_O, NH_3_, CH_4_, CO, and N_2_ chemical soup. Metadynamics were used to analyse the formamide formation from CO and NH_3_ to elucidate possible paths in the potential energy surface. A clever study from the same group was devoted to formamide reaction network in gas-phase and liquid water [111], in which they built up a path of collective variables based on coordination patterns. Summing up the numbers of a row gave the total coordination number for that specific atom. Coordination numbers are not predetermined during the simulation; i.e., path-collective variables are flexible transition mechanisms and unforeseen intermediate states can spontaneously appear during the simulation. More specific details are found in the original papers, while a more general overview is provided in [83]. It should be stressed, however, that these state-of-the-art approaches are hardly applicable by other computational chemists outside the group of developers due to the need to control many technicalities essential to run a physically sensible simulation. As a whole, these approaches are also extremely expensive and need high-performance parallel computer facilities in order to be completed.

To finish this section, some words related to the basis sets are mandatory. Basis sets are functions needed to describe the electrons in a molecular/crystalline system through the definition of molecular orbitals. In general, the most recurrent basis sets are Gaussian-type orbitals (GTOs) and plane waves (PWs). GTOs are localized functions centred on the atoms and accordingly the number of base functions depends uniquely on the number and kind of atoms of the system. In contrast, PWs are periodic functions filling uniformly the space (namely, they are not centred on the nuclei). PWs, due to their periodic nature, are commonly used to describe crystalline systems (at variance with GTOs, which are commonly used in molecular calculations) and the number of PWs depends only on the volume of the crystal unit cell. In real calculations, the molecular orbitals are expanded in a finite number of GTOs (i.e., they are not complete) and, accordingly, GTO-based calculations suffer from the basis set superposition error (BSSE), which heavily affects the structure and energetics of intermolecular and adsorption complexes. For instance, for an adsorbate surface adduct, BSSE artificially overestimates the adsorption energy as the adsorbate exploits the basis set functions of the surface, and vice versa. The counterpoise method developed in the early 1970s is the usual strategy to correct the BSSE [112]. In contrast, PWs do not suffer from BSSE because they uniformly fill the unit cell containing the adsorbate/surface complex. Finally, it deserves to be mentioned that calculations using hybrid functionals are usually only practically done when employing GTOs, as PW calculation of the exact exchange is very expensive. On the other hand, energy and force calculations using GGA are more efficient when PWs are adopted over the GTO basis set.

## 3. Structural Surface Models

There are mainly two different strategies to atomistically model the structure of mineral surfaces: (1) the periodic boundary conditions approach, and (2) the finite cluster approach.

### 3.1. The Periodic Boundary Conditions Approach

**Figure 1:**
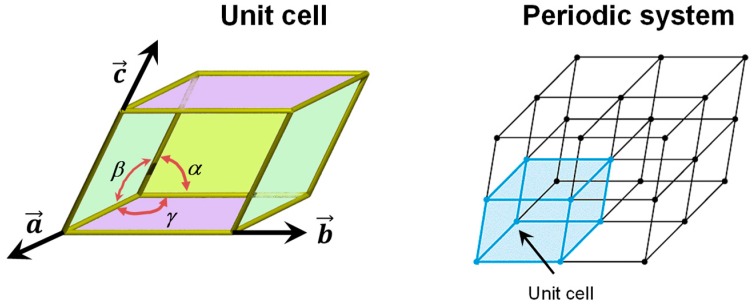
Generic unit cell (including the lattice parameters of ***a***, ***b***, and ***c*** cell vectors and α, β, and γ cell angles) and the periodic system generated by applying the translation symmetry onto the unit cell.

The periodic boundary condition (PBC) approach is usually used to model the bulk structure of crystalline systems; i.e., systems in which its crystallographic unit cell is repeated periodically in the three directions of space. This repetition is done by applying a translation lattice vector ***T*** onto the unit cell (***T*** = m***a*** + n***b*** + p***c***, where ***a***, ***b***, and ***c*** are the cell vectors of the unit cell and m, n, and p are integer numbers), which enforces the translation symmetry of the crystal (see Figure 1). The periodic boundary conditions when applied to the electron density ρ(***r***) of the unit cell ensure that ρ(***r*** + ***T***) = ρ(***r***) for each lattice vector ***T***.

**Figure 2:**
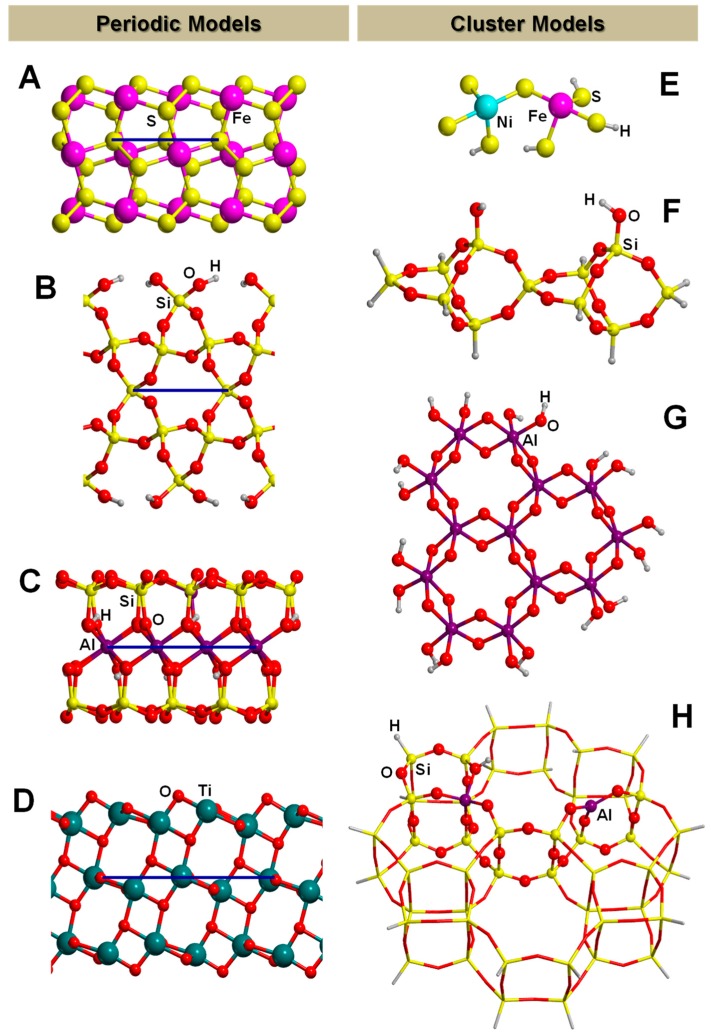
Surface models adopting periodic and cluster approaches. Periodic slab models for the (100) pyrite FeS_2_ surface (**A**), α-quartz hydroxylated (010) surface (**B**), montmorillonite clay (**C**), and anatase (101) TiO_2_ surface (**D**). The unit cell along one direction is shown in blue. Cluster models for surfaces of (Ni,Fe)S vioralite (**E**), silica (**F**), the octahedral sheet of kaolinite (**G**), and an acidic aluminosilicate (**H**). For this latter structure, the atoms represented in balls belong to the high-level zone while the atoms in sticks to the low-level zone in Our Own N-layered Integrated Molecular Orbital and Molecular Mechanics (ONIOM) calculations.

The PBC can also be applied to model crystalline surfaces through the “slab model”, i.e., a finite number of atomic layers parallel to a given Miller (*h*,*k*,*l*) crystalline plane built by cutting out a slice from the corresponding bulk. Examples of slab models of mineral surfaces relevant to the present topic are shown in Figure 2 (A, B, C and D). It is worth mentioning that when PWs are used (see above), due to their intrinsically extended nature, slabs are pseudo 3D systems; that is, they consist of fake periodic 3D systems in which slabs are separated by gaps of empty space large enough to avoid spurious interactions between replicated slabs. In contrast, when localized GTOs are used, slabs are true 2D systems. That is, the PBC conditions only operate in the two directions defining the surface, while the direction perpendicular to the slab becomes non-periodic.

As minerals are materials with ionic and covalent bonds, some caution must be paid when generating slab models by cutting out the bulk. An important factor to take into account is that the slab models have to keep the stoichiometry of the bulk and be charge electroneutral. This means that covalent units must remain untouched; e.g., tetrahedral SiO_4_ units in silicates should not be cut, and that the cations/anions present in the slabs have to balance the total charge to 0. Another aspect to consider is that the slab models cannot exhibit a dipole moment across the surfaces; i.e., they have to be non-polar. Polar surfaces can introduce a catastrophic behaviour in the wave functions, rendering their calculation difficult to converge as a function of the slab thickness. The slab thickness should be such that the corresponding surface energy (*E*_S_) converges with the slab thickness. This quantity represents the energy cost to form the slab from the bulk and can be calculated according to Equation (1):

*E*_S_ = (*E*_slab_ − *NE*_bulk_)/2*A* (1)

where *E*_slab_ is the energy of the unit cell slab, *E*_bulk_ is the energy of the unit cell bulk, *N* is the number of bulk units cells contained in the slab unit cell, and *A* is the surface area, which is multiplied with a factor 2 because the slabs usually exhibit two external surfaces (above and below). Small thicknesses introduce artefacts from geometrical (e.g., excessive slab deformations) and energetic (e.g., inaccurate adsorption energy values) points of view. Since surface adsorption and reactivity is dictated by the structural and energetic features of the surface, different surfaces belonging to the same mineral (identified by different Miller (*hkl*) triplets) may exhibit different chemistry.

### 3.2. The Finite Cluster Approach

This approach consists of extracting from the bulk system a finite (namely, molecular) system, which will be used to simulate the extended system. Figure 2 (structures (E), (F), (G), and (H)) shows different models adopting the cluster approach for mineral surfaces relevant to this topic. The structure of the cluster model must contain the specific site(s) relevant for the chemical problem under study. The underlying idea is that, if this condition is accomplished, one can study adsorption and reactivity without resorting to PBC, but treating the surface as a molecular system. This has an advantage from a methodological point of view: one can simulate surfaces processes with molecular codes, which are much more developed and richer in different methods than the PBC ones. This is particularly appealing when seeking reliable electronic descriptions (e.g., open-shell systems, H-bond interactions), in which highly accurate methods such as CCSD(T) are desirable. The available computer codes dealing with the PBC approach are limited to DFT theory level with the exception of CRYSCOR [113], a program capable of treating electron correlation in solids through local second-order Møller–Plesset perturbation theory (LMP2) adopting Gaussian-type basis functions, and the Vienna Ab initio Simulation Package (VASP) [114], which is based on PW basis set and is capable of evaluating energy at the MP2 level [115]. A second issue concerns the elucidation of complex potential energy surface (PES), such as those involving concerted/synchronic mechanisms or multidimensional reactive paths (the techniques to localize transition states are less developed in PBC codes than in molecular codes).

When one aims to calculate a cluster model at a full QM level, the cluster size is limited by the computational resources, growing steep with the number of electrons. When defining the cluster size, one should also deal with dangling bonds at the edges of the cluster, which should be usually healed by hydrogen atoms, at least for covalent systems. Care should be taken to avoid spurious interactions between the saturating atoms (not present in the original system) and the adsorbate. The limited size of the cluster may be overcome by embedding approaches, popularized by the Our Own N-layered Integrated Molecular Orbital and Molecular Mechanics (ONIOM) methods proposed by Morokuma and co-workers [116]. The main idea is to model the surface with a very large cluster but, to make the simulation feasible, the theoretically description is kept low by using a relatively cheap method. Then, the smaller and most representative region within the large cluster (for instance where the adsorption occurs) is defined and a high-level method is chosen to improve the chemical description of this active region. Energies and forces derived for the two levels and cluster sizes are handled in schemes all derived from the ONIOM approach. Structure of Figure 2H is an example of embedded cluster model, where the atoms represented in balls belong to the high-level zone while those in sticks to the low-level one. For the high-level zone, DFT or post-HF methods are usually used, while for the low-level zone the most recurrent methods are the semi empirical or even the MM ones. Embedded techniques allow for partly solving the “edge effects” problem of the cluster because, since the size of the cluster is large, the edges of the cluster are far from the chemically relevant zone, thus avoiding spurious interactions.

## 4. In Silico Prebiotic Studies on Mineral–Organic Interactions

In the following, we review the quantum mechanical simulations for four classes of minerals: clays, iron sulphides, titanium dioxide, silica and silicates. The selection of this rather limited data set, despite the much higher number of potential minerals available in the Hadeon Eon on planet Earth (vide supra the work by Hazen [36]), is due to the relative paucity of experimental and theoretical studies for different classes of minerals, at least in the context of prebiotic chemistry. We have deliberatly excluded to report studies for ice, despite its extreme importance in the astrochemical context. Note that at the forsterite core of the interstellar grains in the cold molecular clouds reactions between H and O atoms occurred to give water molecules that slowly aggregate in an amorphous ice [14]. This process proceeds up to a point in which a thick mantle of ice is formed and the forsterite core is entirely hidden to extrenal influences. During the mantle growth, other molecules may be sythesized in situ (CO, NH_3_, HCN, etc.) or adsorbed from the interstellar region remaining encapsulated in a form of “dirty ice”. At the surface of the icy mantle and also within its core, due to UV irradiation from nearby stars, many reactions occur, transforming the adsorbed/incapsulated molecules into more elaborated COMs. Nonetheless, the present review focuses on mineral surfaces and not on amorphous ice of the kind found in the grain mantle since it can hardly be classified as a mineral. Indeed, atoms in a mineral are usually held by rather strong covalent and/or ionic forces, while ice is entirely dominated by relatively weak hydrogen bond interactions. Therefore, it is expected that reactive processess at its surfaces may involve large rearrangements of the surface water molecules, as well as proton transfer towards the adsorbed species at variance with the relative rigidity, chemical stability and locality of the catalytic processes at classical mineral surfaces. The interested reader may refer to the excellent recent and broad review on amorphous ice by Hama and Watanabe [26].

### 4.1. Clays

Clays are among the most invoked mineral groups to have played an important role in prebiotic chemical evolution. Even in the first proposal by Bernal, clays were proposed as important mineral materials in prebiotic events.

Clays are aluminium hydrous silicates that exhibit a layered structure engaged by H-bond interactions. One layer is made up by tetrahedral silicate sheets (the unit block being [SiO_4_]) and the other by octahedral hydroxide sheets (the unit block being [AlO_6_], exhibiting OH groups). Clays can be classified as 1:1 (when the clay exhibits a ratio of 1 tetrahedral sheet with 1 octahedral sheet) or 2:1 (1 octahedral sheet is sandwiched by 2 tetrahedral sheets). Kaolinite, dickite, or serpentine belong to the first group, while montmorillonite, nontronite, or saponite belong to the second group. Isomorphic substitutions can take place; usually Al^3+^ replaces Si^4+^ in tetrahedral sheets, and Mg^2+^ replaces Al^3+^ in octahedral sheets. If this is the case, a negative charge per substitution is generated, which is compensated by cations such as Na^+^ or Li^+^ in the interlayer regions. In broken edges (namely, the edge regions in which the tetrahedral and octahedral sheets are truncated), the negative charges are compensated by H^+^, and thus these surfaces are hydroxylated by OH groups.

**Figure 3:**
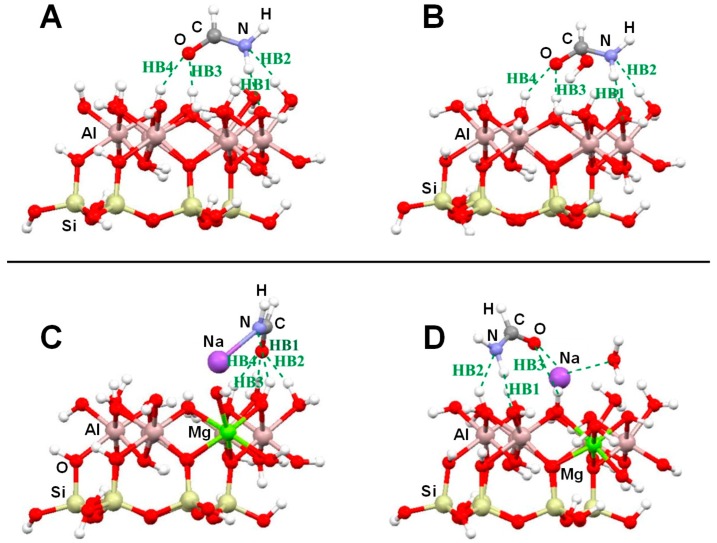
Most stable adducts for formamide interacting with cluster models of the octahedral sheet of kaolinite. (**A**) non-substituted and non-hydrated, (**B**) non-substituted and hydrated, (**C**) substituted and non-hydrated, and (**D**) substituted and hydrated. Adapted from [119].

Several computational works on this topic have already been referenced and summarized in [81]. Some of them were focused on the interaction of formamide (NH_2_CHO, FA) with dickite [117,118] and kaolinite [119,120] surface models, which were simulated adopting cluster approaches. In the cases where no isomorphic substitutions were performed, the driving forces for the interaction were dictated essentially by H-bonding. Indeed, the deformation undergone by FA upon adsorption was due to the formation of the maximum number of H-bond interactions, the C=O and NH_2_ groups acting as H-acceptor and H-donors, respectively. Calculated interaction energies are summarized in Table 1. For the interaction with dickite [117], authors identified that intercalation energies were larger than external surface adsorption energies (−20.2 kcal mol^−1^ and −14.5 kcal mol^−1^, respectively, see Table 1), indicating that the interlayer space provides additional stabilisation due to the higher number of interactions. However, it is worth mentioning that such a comparison could be biased by the excess of flexibility of the cluster models in the intercalation region. On the kaolinite surfaces [119,120], calculated adsorption energies of FA on the non-hydrated octahedral and tetrahedral sheets were found to be −14.8 kcal mol^−1^ and −13.7 kcal mol^−1^ (see Table 1). Simulated IR spectra of FA interacting with kaolinite surfaces were compared with the experimental one [120], which served to conclude that FA indeed interacts through H-bonds, particularly between the Al−OH octahedral kaolinite face and the FA C=O group (see Figure 3A). Temperature programmed desorption (TPD) analysis provided an average binding energy of 11.7±0.24 kcal mol^−1^, which agrees well with the calculated adsorption values. For hydrated octahedral and tetrahedral sheets, the interaction energies decreased to −9.2 (see Figure 3B) and −5.9 kcal mol^−1^, respectively. When isomorphic substitutions were performed and the negative charges compensated by Na^+^, significant changes were identified, both from structural and energetic points of view. When the substitution occurred in the octahedral sheet, a spontaneous H-transfer from the NH_2_ group to one dangling OH group of the surface took place during the optimization, with FA interacting with Na^+^ through its N atom (see Figure 3C) with a very favourable interaction energy of −108.5 kcal mol^−1^. In contrast, in the presence of water molecules solvating the Na^+^ cation, no H-transfer occurred (see Figure 3D) and accordingly the interaction energy dropped to −21.3 kcal mol^−1^. When the isomorphic substitution was in the tetrahedral sheet, the interaction was through the Na^+^ cation and the O atom of FA with an interaction energy of −20.6 kcal mol^−1^, while in the presence of water molecules it was −18.0 kcal mol^−1^.

Aquino et al. [121] studied the interaction of N-methylacetamide with broken clay surfaces modelled by the minimal clusters of (RO)_3_SiOH, [(RO)_3_AlOH]^−^ (R = H, CH_3_, SiH_3_, and Si(OH)_3_), and Si_2_AlO_5_H_3_(OH)_2_. The most remarking aspect is that the interaction of N-methylacetamide enhanced the planarity of the amide bond, increasing the CN double bond character.

**Figure 4:**
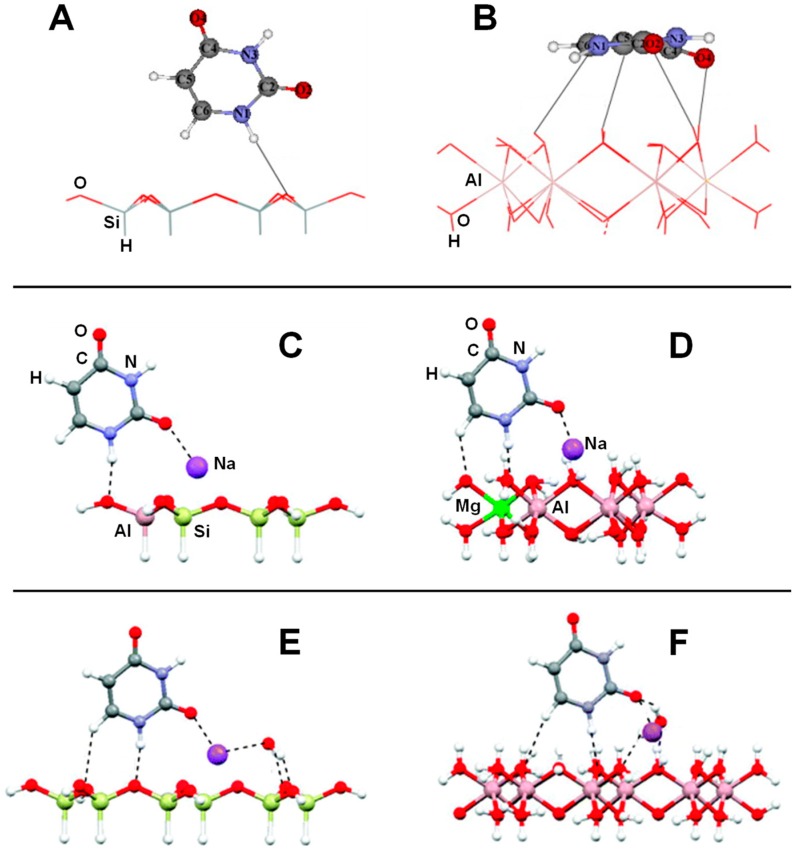
Most stable adducts of uracil interacting with the dickite clay through the tetrahedral (**A**) and octahedral (**B**) sheets and with Na^+^-kaolinite through non-hydrated tetrahedral (**C**) and octahedral (**D**) sheets and in the presence of one water molecule ((**E**,**F**), respectively). Adapted from [122] and [123].

Another set of interesting recent works are those related to the interaction of nucleobases with clays. Leszczynski and co-workers studied the interaction of thymine (T) and uracil (U) with cluster models of dickite [122] and kaolinite [123], in which the nucleobase interactions were described for the tetrahedral and octahedral sheets separately. Calculated adsorption energies are summarized in Table 2. On dickite clusters without isomorphic substitutions, the interactions were essentially based on H-bonds. As occurred for the FA case, U and T were more favourably adsorbed on the octahedral sheets than on the tetrahedral ones (e.g., for the U case, −30.3 and −3.6 kcal mol^−1^, respectively) because of the formation of more and/or stronger H-bonds (see Figure 4A,B). The same explanation was used to interpret why the interaction of U was found to be more favourable than T (e.g., on the octahedral sheet the values were −30.3 and −21.2 kcal mol^−1^, respectively). Generally, the distinctive methyl group in T did not significantly affect the interaction compared with U. However, calculations were carried out at B3LYP/6-31G(d) level of calculation, missing dispersion interactions. If they were accounted for, the energy differences between U and T would be probably different. The presence of an explicit water molecule at the interface region induced a stabilisation of the complexes, probably due to H-bond cooperative effects. The most favourable complex was for U adsorbed on a hydrated octahedral dickite fragment (−47.8 kcal mol^−1^, see Table 2). Different structural and energetic features were found when U and T interactions occurred on an isomorphic substituted Na^+^-kaolinite cluster models [123]. For these cases, the adsorption was dictated by electrostatic interactions between the surface Na^+^ cation and the O atoms of U/T plus H-bonds between the NH groups of U/T (acting as H-bond donors) and the kaolinite O surface atoms (see Figure 4C,D). Due to the electrostatic interactions, the adsorption energies were significantly larger and more negative than in non-substituted dickite (see Table 2). Calculations were carried out both at M05-2X and B3LYP DFT levels of theory, in which M05-2X adsorption energies were systematically larger than the B3LYP ones, probably because the former partly accounts for dispersion interactions. Despite this, the adsorption energies for U and T were found to be similar; e.g., on the octahedral Na^+^-kaolinite fragment the energies were −46.1 and −44.4 kcal mol^−1^, respectively, at M05-2X (see Table 2). Once again, on the octahedral sheets, the interaction was significantly more favourable than on the tetrahedral ones (e.g., for the U case, −46.1 and −31.0 kcal mol^−1^, respectively, at M05-2X). The presence of one explicit water molecule interacting with the Na^+^ cation (see Figure 4E,F) exerted in general a small destabilization (between 1 and 3 kcal mol^−1^) of the complexes, due to a screening effect onto the charge of the cation.

**Figure 5:**
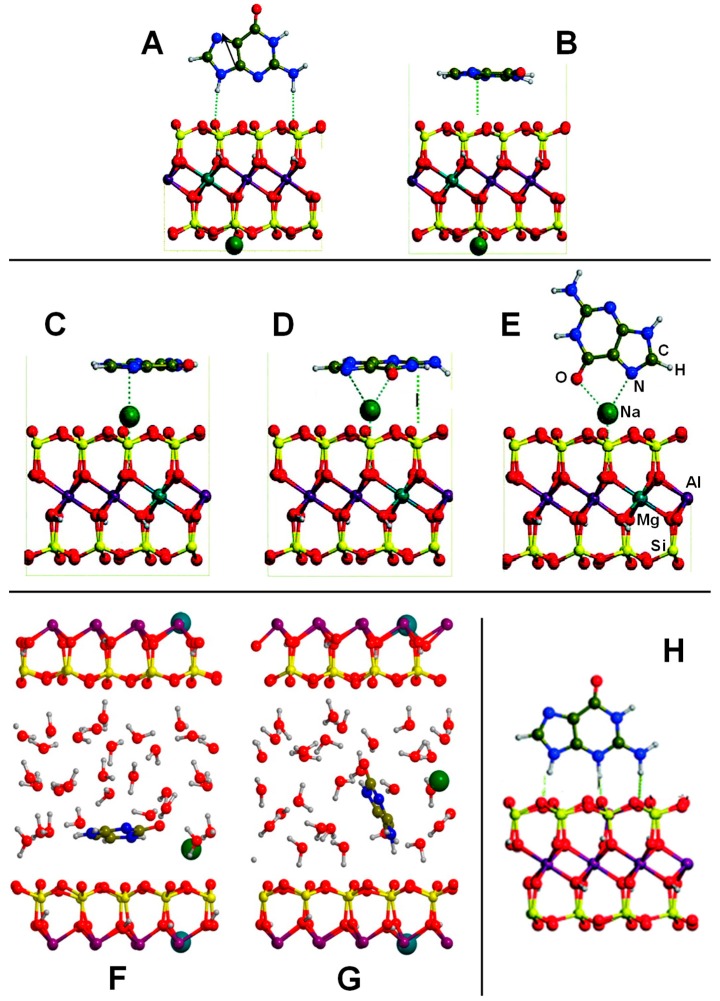
Most stable adducts of guanine interacting with Na^+^-montmorillonite through the Na^+^-free side in non-hydrated conditions in a perpendicular (**A**) and parallel (**B**) way, through the Na^+^-containing side in non-hydrated conditions adopting cation–π/ring (**C**), cation–π/displaced (**D**), and cation–heteroatom interactions (adapted from [124]). Representative structures for cytosine interacting with Na^+^-montmorillonite in hydrated conditions ((**F**,**G**), adapted from [125]). The most stable adduct of guanine interacting with an acidic external surface of montmorillonite ((**H**), adapted from [126]).

Mignon and co-workers also studied the interaction of nucleobases with clays, but on the external surfaces of Na^+^-montmorillonite [124], also considering hydration [125], and on the acidic external surfaces of montorillonite [126] (i.e., H^+^ as counterion). All these calculations were performed adopting a PW-based periodic approach at a PBE-D2 level. Since montmorillonite is a 2:1 clay, nucleobase interactions were studied considering only the tetrahedral layers. For Na^+^-montmorillonite, authors performed an exhaustive exploration of the different complexes considering parallel and orthogonal orientations with respect to the external surfaces [124]. On the Na^+^-free side, since dispersion forces constituted almost the total contribution to the adsorption energy, in most of the cases, nucleobase adsorption in a parallel orientation was found to be more favourable than the orthogonal one (see Figure 5A,B), with calculated adsorption energies ranging from −4 to −11 kcal mol^−1^ (see Table 3). On the Na^+^-containing side, different adsorption modes were identified: (1) classical cation–π/ring interactions (i.e., the electrostatic interaction between Na^+^ and the electric quadrupole of the aromatic rings, see Figure 5C), (2) cation–π/displaced interactions (i.e., electrostatic interaction between Na^+^ and the electric quadrupole of the exocyclic heteroatoms, see Figure 5D), and (3) cation–heteroatom interactions (i.e., electrostatic interaction between Na^+^ and the lone pairs of the heteroatoms, see Figure 5E). The two former interactions imposed parallel adsorptions, while the latter orthogonal ones, allowing for the formation of efficient H-bonds. Calculated adsorption energies (see Table 3) indicated that cation–π/ring interactions were less favourable than the other two interaction types due to the smaller contributions of the electrostatic interactions. On the other side, cation–heteroatom interactions were found to be more favourable than the cation–π/displaced ones for guanine and cytosine (−27.6 and −27.0 vs. −26.1 and −26.6 kcal mol^−1^, respectively), as they presented a bidentate coordination (hence enhancing the electrostatic contribution), while for adenosine, uracil and thymine, the cation–π/displaced configurations were more favourable. For the particular case of cytosine on the Na^+^-containing face, hydration effects (simulated by the presence of 24 water molecules in the interlayer region) were also studied by the same authors by means of AIMD simulations [125]. When cytosine was close to the external face, it remained adsorbed on the surface in a parallel orientation (see Figure 5F), as it was stabilized by dispersion interactions. Additionally, the cytosine O heteroatom established a cation–heteroatom interaction with Na^+^, which remained adsorbed on the surface and partly solvated by water molecules. The cytosine NH group also established H-bond interactions with water. In contrast, when cytosine was away from the surface, it adopted an orthogonal orientation interacting with O surface atoms through H-bonds and with Na^+^ (desorbed from the surface) through cation–π/displaced interactions (see Figure 5G). Such a Na^+^ desorption was in agreement to what was found by the same authors in the hydration of Na^+^-montmorillonite, simulating a swelling process [127].

The adsorption of adenine, guanine, and cytosine on acidic montmorillonite surfaces in dry conditions was also studied [126], in which both the octahedral and tetrahedral substituted forms were considered, as they have different acidic properties. Both parallel and orthogonal orientations were considered. In almost all the complexes, a spontaneous transfer of the acidic proton from the surface to the nucleobases was observed (see Figure 5H), showing the strong acidity of the external surfaces. Adsorptions were found to be more favourable in the octahedral substituted forms (by about 10 kcal mol^−1^) than in the tetrahedral ones (see Table 3), indicating a larger Brønsted acidity in the former systems. Nucleobase adsorptions were dictated by H-bond interactions (nucleobases acting as H-bond donors toward the surface) and dispersion interactions (of great significance in the parallel orientations), as well as by electrostatic interactions between the positively charged nucleobases and the negatively charged surfaces. The most stable adducts gave adsorption energies of −49.4, −50.0 and −44.4 kcal mol^−1^ for adenine, guanine, and cytosine, respectively (see Table 3), ca. twice the adsorption values when interacting with Na^+^-montmorillonite. Authors identified that such a difference is directly correlated to the larger proton affinity of these nucleobases compared to their cation affinities.

To the best of our knowledge, only one theoretical work dealing with the interaction of amino acids with clays is available. It concerns the adsorption of glycine on K^+^-montmorillonite surfaces, both in dry and hydrous conditions [128]. The structural model of the clay was based on a periodic approach and the static calculations were performed with PBE-D2/PWs and numerical atomic orbitals, with both basis sets providing very similar results. Under strict dry conditions, optimization of glycine (Gly) placed in the interlayer region resulted in a spontaneous transformation from its canonical form (NH_2_CH_2_COOH) to the zwitterionic one (NH_3_^+^CH_2_COO^−^), the final adsorption energy being about −20 kcal mol^−1^. In the optimized structure, the COO^−^ group interacted with the K^+^ cation while the NH_3_^+^ group with the basal tetrahedral O atoms of the surface. These interactions were responsible of the stabilization of the Gly zwitterion, rendering the clay a solid solvent. Similar Gly adsorption assessments were carried out accounting for different levels of hydration in the interlayer region. For all the cases, the zwitterion configuration was the most stable one, with adsorption energies of −38, −43, and −47 kcal mol^−1^ in the presence of 8, 12, and 20 water molecules, respectively. Interestingly, in all the optimized structures for these hydrated cases, the K^+^ cation moved to the middle of the interlayer region, far from the surface, fully solvated by the confined water molecules. Finally, the replacement of the K^+^ counterion by glycinium (i.e., the protonated form of glycine, NH_3_^+^CH_2_COOH) was found to be moderately favourable, which allowed the authors to explain the experimental findings that detected glycine adsorption in montmorillonite in the form of glycinium cation [129].

### 4.2. Iron Sulphides

As mentioned in the Introduction section, the presence of iron sulphides as key materials in prebiotic chemical evolution was postulated first in the chemoautotrophy theory developed by Wächtershäuer [40] and Russell et al. [130], who were inspired by the discovery of a unique ecosystem in the hydrothermal deep sea vents (black smokers) by the Alvin submarine, indicating that life is possible without the support of photosynthetic processes. This theory advocates the role of iron sulphide surfaces as active catalysts promoting the growth of organic superstructures through C fixation, which is driven by redox processes. The pioneering idea of the iron–sulphur world theory is that the chemical evolution begun as an autocatalytic surface metabolism, in which formed organic products serve as ligands for activating the catalytic centres whence they arose. The energy supply of this surface metabolism is provided by the redox energy of the oxidation of iron sulphide (FeS) to pyrite (FeS_2_) and the reduction of H_2_S to form H_2_ (ΔG = −38.6 kJ mol^−1^) [131]. This energy could have initially been used to convert small molecules (e.g., ammonia and carboxylic acids) into more complex forms as a first step towards a primordial metabolism. On the early Earth such a scenario could have operated in hydrothermal vents at high pressure and temperature. The validity of this theory has been demonstrated by several experiments: formation of carboxylic acids from organic sulphides (e.g., CH_3_SH) and CO on (Fe,Ni)S surfaces [42,132], conversion of N_2_ to NH_3_ on FeS in the presence of H_2_S [133], formation of alanine and other amino acids by reaction between NH_3_ and pyruvate on (Fe,Ni)S surfaces [134], formation of amino acids and hydroxyl acids from (Fe,Ni)S surface-bound cyano and methylthio ligands in the presence of CO [135], and activation of amino acids to form peptides in the presence of CO and H_2_S/CH_3_SH on (Fe,Ni)S surfaces [136,137].

A first quantum chemical assessment of the viability of the C fixation cycle was carried out by Leszczynski and co-workers [138]. By means of a minimal cluster model for a (Fe,Ni)S surface (shown in Figure 2E), they studied at the B3LYP/TZVP level of theory the thermodynamics of the individual reactions of the surface metabolism cycle leading to the production of acetic acid from CH_3_SH, CO and H_2_O. Free Gibbs energies of reactions were obtained by applying thermochemical corrections on the optimized stationary points (i.e., reactants, products and intermediates) at 373.15 K. Results indicated that the overall reaction was endergonic by 16.7 kcal mol^−1^. Despite this, the crucial step in which FeS_2_ forms by reaction of FeS and H_2_S was found to be slightly exergonic by −2.9 kcal mol^−1^, indicating that this step can operate as an initial energy source for other primordial surface metabolic reactions. It is worth mentioning that these results were obtained by introducing temperature effects in an approximate way (i.e., through thermochemical corrections) and that the effects of the high pressure were not accounted for, which can be of great relevance (see below). Furthermore, the thermodynamic of these reactions may be very dependent on the level of calculations and more refined approaches are needed, also enlarging the cluster or adopting PBC. Accordingly, a more rigorous treatment of the extreme conditions (e.g., by means of AIMD simulations) could give different results. Additionally, the reaction kinetics were not explored and still remain to be investigated. Hydration effects were also omitted, which are expected to play an important role under extreme conditions (see below).

**Figure 6:**
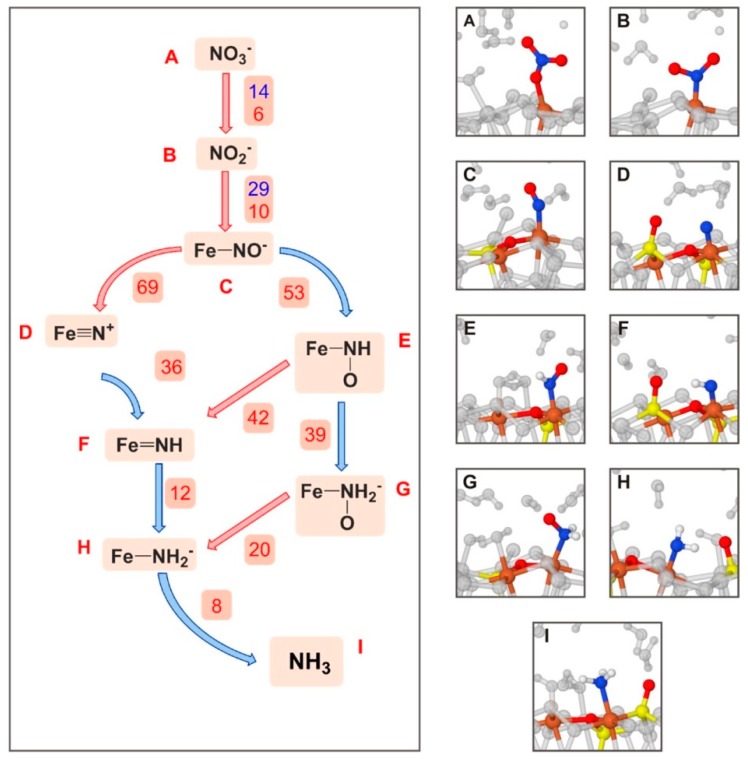
PBE-D2/PW chemical reaction network for the NO_3_^−^ → NH_3_ conversion on the pyrite surface under hot-pressurized conditions. Red arrows refer to oxygen transfers, while blue arrows to hydrogen transfers. Activation free energies are in kcal/mol. Blue values refer to barriers obtained on perfect pyrite surface, whereas red values correspond to the defective surface. The representative snapshots of the reactant, intermediate, and product species are also shown. Adapted from [139].

Stirling and co-workers [139] investigated at PBE-D2/PWs the first steps of the surface metabolism by simulating the formation of NH_3_ from NO_3_^−^ on sulphur vacancy-defective (100) FeS_2_ surfaces under hydrothermal conditions; i.e., a hot-pressurized water environment. Simulations were carried out by means of ab initio metadynamics calculations. The main conclusion of the work was that the NO_3_^−^ → NH_3_ transformation on the pyrite surface was found to be energetically feasible through a network mechanism (shown in Figure 6), in which at each step atom transfers (either O or H) took place. The bottleneck of the process was the reduction of NO^−^. It was found that the role of pyrite was twofold: it was a reactant by abstracting O atoms in the initial steps, and it bound the N-containing species by keeping the reactive species in simultaneous contact. In addition, the role of water was found to be twofold: it was a reactant, and it formed part of the environment supplying the favourable thermodynamic conditions. This latter aspect is of vital importance for the occurrence of the processes. Indeed, under extreme conditions, pyrite surface is hardly covered by water [140], thus allowing for the NO_3_^−^ adsorption and its subsequent reduction. Moreover, the same processes were calculated by means of static calculations (i.e., 0 K and 0 bars) and in the absence of water showed much slower reactions, thus indicating that the extreme conditions induce remarkable reaction acceleration effects.

A set of very important computational works belonging to the iron–sulphur world are those published by Marx and co-workers (references are provided along the subsection) adopting AIMD at the PBE/PW level. In these studies, the interaction of Gly and its activation toward peptide bond formation on periodic models of both clean and sulphur vacancy-defective (100) pyrite surfaces were investigated by means of ab initio molecular dynamics/metadynamics simulations at 500 K and 20 MPa (resembling the extreme conditions of the hydrothermal environments).

**Figure 7:**
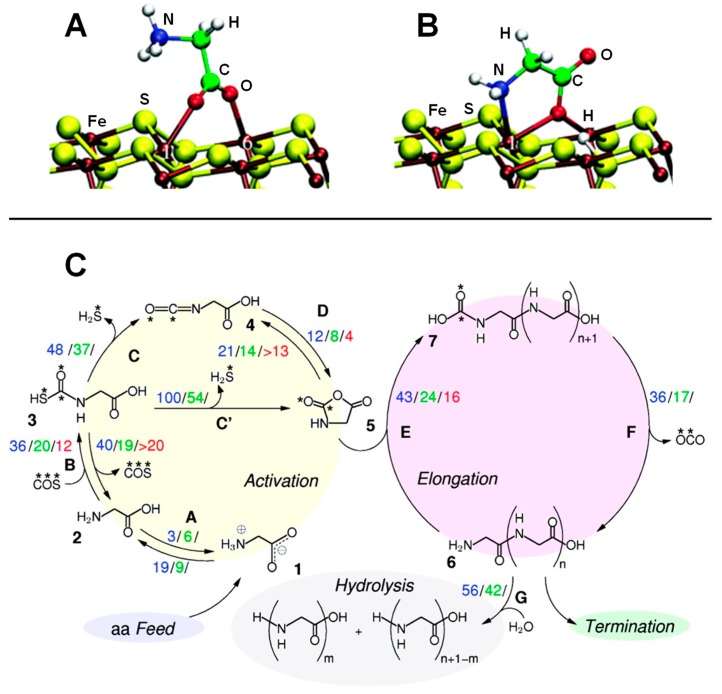
(**A**,**B**) Stable adducts for the adsorption of glycine on a sulphur vacancy-defective (100) FeS_2_ surface in hot-pressurized water conditions at the PBE/PW level. Adapted from [142]. (**C**): Peptide synthesis cycle via activation of glycine into N-carboxyanhydride (NCA) and the subsequent condensation reaction. Hydrolysis of the peptide form was also considered. Calculated free energy barriers are reported in units of k_B_T for the sake of comparison between the three conditions considered: ambient bulk water (blue); pyrite-free hot-pressurized water (green); and pyrite-interfacial hot-pressurized water (red). Adapted from [143].

In aqueous solution in normal conditions, the stable form of Gly is the zwitterionic one. On the clean, non-defective pyrite surface two possible adsorption states were identified in the pyrite-water interface [140,141]: one exhibiting a monodentate adsorption mode via one carboxylate oxygen, the other adopting a bidentate (O,O) adsorption mode. The relative attachment strength of these two adducts in hot-pressurized water conditions was determined by executing AIMD simulations. It was found that the monodentate adduct desorbed readily on a picosecond time-scale in a water-mediated process; i.e., a water molecule “attacked” via H-bond the O atoms responsible of the surface binding. The bidentate (O,O) adduct exhibited larger retention times, in which the two O atoms temporarily detached from the surface but not both at the same time. On the sulphur vacancy-defective pyrite surface, two plausible adducts were identified [142]. One involved the zwitterionic Gly adopting a bidentate (O,O) adsorption mode (see Figure 7A). In the other, the COOH acidic proton was transferred to the surface allowing a (N,O) adsorption mode (cyclic structure, see Figure 7B). AIMD simulations indicated that both adducts were actually stable at the hot-pressurized water/pyrite interface. Gly was found to be much more strongly adsorbed on the defective surface than on the ideal surface, as a result of strong Fe coordination at the point defect. Additionally, authors investigated the paths leading to the full desorption of the two glycine adducts using the metadynamics technique. Results indicated that to reach a complete Gly desorption several steps were required. Calculated free energy surfaces showed that the final desorbed Gly (becoming fully solvated) was much more stable than on the surface (about 48 kcal mol^−1^), and the free energy barriers associated with the desorption process were found to be of 21.5 kcal mol^−1^, which can roughly be translated into retention times of the order of milliseconds.

An interesting point to check was whether these retention times were long enough to allow for the formation of peptides on the surface. This question was addressed at the PBE/PW AIMD level by Schreiner et al. [143] and Nair et al. [144]. In these works, formation of peptides on clean and defective pyrite surfaces was investigated considering both normal and extreme conditions by means of ab initio metadynamics simulations. The condensation of two Gly molecules was done following the activation of one of them in the form of N-carboxyanhydride (NCA), as it presents an activated CO group [145,146,147]. It is worth mentioning that formation of NCA is possible by reaction of Gly with COS, followed by H_2_S elimination and an intramolecular cyclization (see Figure 7C, activation route). However, the addition of COS onto Gly is only possible when this latter is in its canonical form, which is not stable at normal water conditions. Interestingly, the amino acid canonical form is stabilized in hot pressurized water since the water dielectric constant is dramatically reduced to about 6 [148]. The simulated reaction steps (i.e., Gly activation, elongation, and hydrolyses) are sketched in Figure 7C. They were calculated in ambient bulk water (ABW), in pyrite-free hot-pressurized water (HPW) and some steps in pyrite-interfacial hot-pressurized water (PIW). For the sake of comparison between these conditions, authors reported the computed free energy barriers in units of k_B_T. According to these values, formation of NCA was highly enhanced when going from ABW to PIW (the free energy barriers became progressively significantly reduced), indicating that both the hot-pressurized water and the pyrite interface played a catalytic role. The role of the hot-pressurized water was, as mentioned, to stabilize the Gly canonical form to further react with COS. The essential catalytic effect of pyrite was reducing the entropic contribution to the barriers by immobilizing the reactive species. Interestingly, it was found that the estimated Gly retention times (see above) were long enough to allow the occurrence of the reactions, particularly in the defective pyrite surfaces. Formation of the peptide (see 7C, elongation route) started from NCA reacting with glycine, which once again must be in its neutral form. This route was also found to be accelerated in extreme conditions (i.e., free energy barriers of 43 k_B_T, 24 k_B_T and 16 k_B_T in ABW, HPW, and PIW, respectively) due to both the high temperature and the surface immobilization. To have a complete picture of the peptide formation and related processes, the peptide hydrolysis was also simulated [143,149]. Hydrolysis was found to have higher free energy barriers compared to those of the peptide formation (see Figure 7C, hydrolysis route), thus indicating that peptide elongation cycles dominate over hydrolysis.

Marx and co-workers have also studied if nanoconfinement effects can play any role in the iron–sulphur world scenario. The underlying idea is that nanoconfined liquids (i.e., liquids confined in nanometric spaces) exhibit very different structural and dynamic properties compared with those of the corresponding bulk systems, which in turn can deeply affect chemical reactions taking place in nano-solvation regimes. In a set of AIMD-based works, the properties of nanoconfined water lying between two (001) terminated sheets of mackinawite (FeS) at extreme conditions [150,151] were investigated. More interestingly, Muñoz–Santiburcio [152], using ab initio metadynamics PBE/PWs simulations, studied the peptide formation routes in hot-pressurized nanoconfined water intercalated between two FeS layers, whose mechanisms and energetics were compared with that in bulk water at the same conditions. Authors established a complex network of chemical reactions, indicating that nanoconfinement induced a richer chemistry than bulk HPW. Factors favouring one path or the other were identified to be related to steric factors (as geometry orientation is constrained by the small space) as well as to the ability of nanoconfined interfacial water to stabilize charged species. The authors demonstrated that in nanoconfined water at extreme conditions it is possible to achieve thermal activation for reactions involving charged intermediate species, while such stabilization is not possible in bulk HPW, in which only thermal activation of neutral species is possible.

### 4.3. Titanium Dioxide

According to the mineral evolution theory [153,154], which outlines the diversification and increase of complexity of minerals in planets and moons, TiO_2_ on early Earth is postulated to have been present (although as a minor phase) in the first stage of mineral evolution (i.e., in Eoarchaen, ≈ 4.0–3.6 Ga) in the form of mineral deposits in most igneous rocks and sediments formed by solidification of cooled magma. The presence of TiO_2_ minerals in this stage is not surprising, considering that they are building blocks detected in stardust nucleation/condensation regions forming interstellar pre-solar dust grains and that they have been found in different meteorites [155,156] and in zones of ancient asteroid impacts [157]. The relatively low abundance of TiO_2_ on the Earth’s crust (around 1%) is not detrimental of its role in prebiotic processes as a catalyst. Indeed, TiO_2_ is an excellent catalyst, as widely demonstrated in different industrial and technological applications [158,159]. There are three naturally-occurring TiO_2_ crystalline polymorphs: rutile, anatase and brookite. The two formers phases are the most studied ones, both in terms of fundamental physico-chemical properties and for technological applications. The rutile (110) and the anatase (101) surfaces are the most stable crystal faces and dominate the crystal morphologies. In both surfaces, the most exposed Ti atoms are penta-coordinated (acting as a Lewis centre) while O atoms can act as strong H-bond acceptors up to host H^+^ from surface-induced deprotonation processes. Therefore, the TiO_2_ surfaces exhibit both Lewis acidic and Brønsted basic sites.

Ojmäe et al. [160] studied the adsorption of different carboxylic acid-containing molecules, including Gly, on rutile TiO_2_ nanoparticles using spectroscopic techniques (FTIR, Raman, powder XRD, and TEM) complemented by PW-based periodic quantum mechanical simulations using the PW91 DFT method, in which the adsorptions were simulated on the rutile (110) surface. Calculations indicated as the most stable adduct Gly adsorbed in zwitterionic form, in which the O atoms of the carboxyl group attached to the surface Ti atoms, while the NH_3_^+^ group established two strong H-bonds (distances of 1.3–1.4 Å) with bivalent surface O atoms. The calculated adsorption energy for this adduct was −47.1 kcal mol^−1^ (see Table 4). The other explored adduct presented Gly in its deprotonated form, about 9 kcal mol^−1^ more unstable (see Table 4). Such an energy difference was caused by the strong H-bond interactions of the NH_3_^+^ group with the surface in the zwitterionic adduct, which were absent in the deprotonated one. Comparison with the spectroscopic data was not possible since Gly was not identified to be adsorbed on the surface because, according to the authors, the amino acid preferred to remain solvated in water solution. These experimental findings were in line with the simulations carried out by Langel et al., [161] in which the adsorption of Gly (as well as methionine, serine and cysteine) on partially hydroxylated rutile (110) and (100) surfaces was investigated in wet conditions (modelled with the presence of 16 H_2_O molecules) using PW-based AIMD simulations. Results indeed indicated that binding of the carboxylate groups to the surface through hydrogen bonds and Ti–OH interactions were weak in all cases.

The relative stability between the zwitterionic and the deprotonated adducts were reversed in the work of Tonner [162], who studied the interaction of Gly and proline on the rutile (110) surface using PW periodic simulations at the PBE level of theory. In this work, the deprotonated adduct was found to be more stable than the zwitterionic one by 2.2 kcal mol^−1^ (see Table 4). The reason for the discrepancy between the results of Ojmaë and Tonner is that in the latter a new and more stable deprotonated adduct was found. Scanning tunnelling microscopy [163] and photoelectron diffraction [164] experiments confirmed that glycine adsorbed as a deprotonated form on the rutile (110) surface in detriment of the zwitterionic one. Photoemission and near edge X-ray absorption fine structure (NEXAFS) spectroscopy dedicated to study the adsorption of phenylalanine on the rutile (110) surface also pointed out to a deprotonated form of the amino acid [165].

The adsorption of proline was also found to occur in its deprotonated state, the energy difference with the zwitterionic one being larger than for the Gly case (i.e., 4.5 kcal mol^−1^, see Table 4) [162]. Adsorption of cysteine was also identified to be in its deprotonated state, but the –SH group of the side chain became also deprotonated upon adsorption [166] (in agreement with AIMD simulations of Langel et al. [161]). The competitive zwitterionic adduct to this complex also exhibited –SH deprotonated, lying 2.8 kcal mol^−1^ high in energy than the most stable one (see Table 4). The adsorption of aspartic acid on rutile was investigated by Hazen and co-workers [167] combining potentiometric titrations and batch adsorption experiments with quantum chemical simulations. To reconcile theory with experiment, authors deduced that two reaction stoichiometries were operating. At low amino acid surface coverages, aspartic acid adsorbed in a (O,O) fashion. At high surface coverages, adsorbed aspartate species belonged to the inner adsorption sphere interacted with incoming amino acids, thus forming hydrogen bonded aspartate surface species. Unfortunately, no adsorption energies were reported in the work.

**Figure 8:**
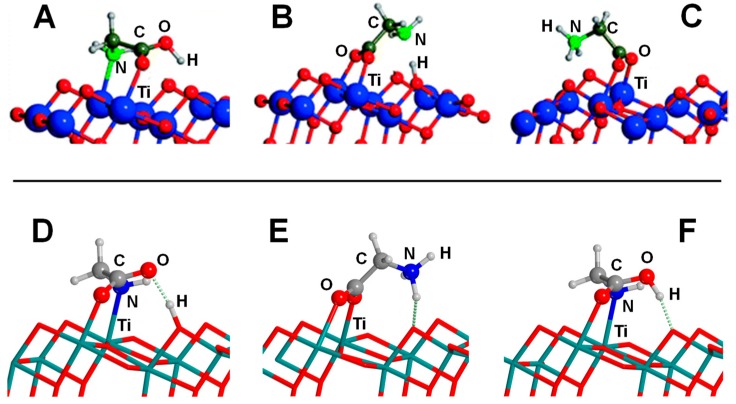
Summary of the most stable adducts formed for glycine adsorption on the anatase (101) surface. (**A**–**C**) are the canonical, deprotonated and zwitterionic forms found in [140], while (**D**–**F**) are those found in [141].

Adsorption of Gly on the anatase (101) surface has also been reported. Szieberth et al. [168], by means of periodic calculations at the PBE0 hybrid DFT level using GTOs as basis functions, found the canonical form of Gly as the preferred adsorption state adopting a (N,O) adsorption mode plus a H-bond with the carboxylic OH group (see Figure 8A). This structure was close in energy to the deprotonated structure (1.1 kcal mol^−1^ higher in energy), adopting a bidentate (O,O) adsorption mode, while the zwitterionic form was found to be 9.1 kcal mol^−1^ more unstable (see Figure 8B,C, respectively, and Table 4 for adsorption energy values). However, in a recent work, Pantaleone et al. [169], by means of periodic PBE/PW-based simulations, identified a (N,O) deprotonated adduct as the most stable one (Figure 8D), followed by the zwitterionic (Figure 8E) and the canonical (Figure 8F) adducts, these two latter structures being 1.9 and 2.5 kcal mol^−1^ higher in energy than the deprotonated adduct, respectively (see Table 4). Since the energy differences between these adducts are small, in this latter work, AIMD simulations were also carried out to analyse whether temperature effects could influence the stability of these structures. Results indicated that the canonical structure converted to the deprotonated one, highlighting the relevance of dynamic effects, which cannot be ignored when the relative energy of specific adducts are very close.

Pantaleone and co-workers extended the study up to 10 different amino acids, i.e., Leu, Met, Phe, Ser, Cys, Glu, Gln, Lys, His, and Arg, therefore covering many different amino acidic functionalities. Results indicated that the relative stability between the deprotonated and the zwitterionic adducts depends on the amino acid side chain due to a delicate balance between favourable interactions and steric hindrances of the side chain with the surface. For those cases in which the side chain could not establish strong interactions (i.e., Leu, Met, Phe, Ser, Cys, and Arg), steric hindrances destabilized the deprotonated complexes favouring the zwitterionic one. In contrast, for those amino acids in which the lateral chain interactions were strong enough to overcome the destabilization caused by the steric hindrance (i.e., Glu, Gln, Lys, His), the deprotonated forms were the most stable ones. For the Arg case, Li et al. [170] studied different conformations on the anatase (101) surface, without considering the zwitterionic adduct; therefore, they did not compare deprotonated vs. zwitterionic forms.

One remarkable work dealing with a prebiotic processes is that reported by Civiš et al. [171], in which they investigated the synthesis of sugars from formaldehyde combining experimental and theoretical data. Experiments found that formaldehyde (H_2_CO) treated with laser pulses in the presence of anatase led to the formation of several sugars, i.e., glycolaldehyde, threose, arabinose, ribose, xylose, glycerol, and diglycolic acid. Remarkably, the synergy between the catalytic role of TiO_2_ and light was highlighted, as irradiation experiments in the absence of TiO_2_ or non-irradiated sample experiments in the presence of TiO_2_, gave far less sugar variability in the final sample. DFT calculations were employed to give an atomistic interpretation of the experimental observations using PBE/PW periodic simulations. The anatase surface was modelled with the O-vacancy defective (001) surface, giving a triplet state due to the presence of two neighbouring undercoordinated Ti^3+^ ions, each one containing one unpaired electron with the same spin. A potential energy surface was calculated to provide a plausible mechanism for the formation of glycolaldehyde (HOCH_2_CHO) from two H_2_CO molecules, which consisted of two steps: (1) adsorption of the two H_2_CO molecules on the Ti^3+^ cations (favourable by about −45 kcal mol^−1^), and (2) C–C coupling between the two H_2_CO molecules, followed by an H-transfer from one moiety to the other to form HOCH_2_CHO (with an energy barrier of about 27 kcal mol^−1^). Authors highlighted that the presence of O-vacancies is crucial for the catalytic activity since the unpaired electrons on the Ti^3+^ cations enabled the C–C bond formation and subsequent H transfer.

**Figure 9:**
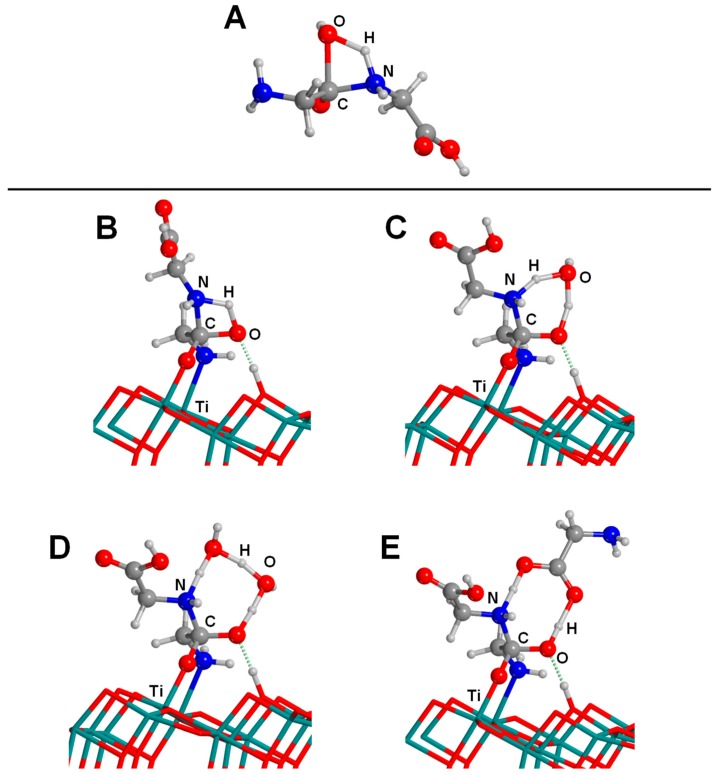
Transition state structures for the peptide bond formation between two glycine molecules: (**A**) uncatalyzed gas-phase process; (**B**) in the presence of the anatase (101) surface; (**C**,**D**) in the presence of the anatase (101) surface assisted by one and two water molecules, respectively; and (**E**) in the presence of the anatase (101) surface assisted by a third glycine molecule. Adapted from [172].

In recent work, Pantaleone et al. [172] focused on the peptide bond formation between two Gly molecules on the anatase (101) surface. The study was carried out at the PBE/PW theory level adopting a periodic approach. As mentioned in the Introduction section, the peptide bond formation is a condensation reaction, therefore facing the water paradox; i.e., it is thermodynamically unfavourable in water conditions. Additionally, the uncatalyzed gas-phase reaction is concerted, involving a nucleophilic attack of the N atom of the amino group toward the C atom of the carboxylic group followed by water elimination due to H transfer from the NH_2_ group to the OH one (see Figure 9A), with an associated free energy barrier at T = 298 K of 44–55 kcal mol^−1^, as computed in [173,174,175,176]. Pantaleone and co-workers addressed the role of the TiO_2_ surface in lowering the kinetic barrier and thermodynamically favouring the reaction. All the studied reactions started with the most stable deprotonated adduct of Gly adsorbed on the anatase (101) surface.

When a second Gly reacted with the adsorbed one, the peptide bond formation reaction followed a stepwise mechanism, in which the nucleophilic attack and the water formation split in two steps. The free energy barrier at 298 K associated with the nucleophilic attack (see Figure 9B) was reduced to 35.6 kcal mol^−1^ (a lowering of 6 kcal mol^−1^ compared with the gas-phase reaction), showing some catalytic effect of the surface. Authors also considered the presence of water molecules (hence mimicking moderately dry conditions) assisting the H-transfer process, which indeed significantly reduced the barrier to 20.7 and 8.0 kcal mol^−1^ with one and two water assistant molecules, respectively (see Figure 9C,D). Thus, the bottlenecks of the reaction appeared to be the formation of water, with free energy barriers of 23 and 18.3 kcal mol^−1^, respectively. Moreover, the presence of a third Gly molecule acting as H-transfer assistant was also studied. The aim here was to provide an atomistic interpretation of the experiments by Martra et al. [57], who reported the catalytic polymerization of glycine monomers on TiO_2_ surfaces by successive feeding of the monomers under strict gas-phase conditions up to the 15-Gly-mers. The nucleophilic attack presented a very low free energy barrier (2 kcal mol^−1^) due to the actual catalytic effect exerted by the third Gly molecule. Here, Gly assisted the H-transfer using both the O atom of the C=O and the H atom of the OH carboxyilic groups (see Figure 9E). As a final outcome, it was found that the TiO_2_ surface also favoured the thermodynamics of the reactions because the released water directly attached undercoordinated surface Ti atoms through stable covalent dative bonds.

### 4.4. Silica and Silicates

Silica and silicates materials are abundant minerals constituting not only the Earth crust but also the nuclei of meteorites, comets and interstellar dust particles. Therefore, it is worth studying the interaction of organic compounds and the evolution of prebiotic processes with these naturally-occurring materials.

Concerning pure silica (SiO_2_) phases, an extended review [82] has recently been published. Accordingly, here we only focus on new works. Bulk SiO_2_ consists of tetrahedral units of [SiO_4_] that are connected through their corners forming a wide set of structures and polymorphs. Pure silica surfaces exhibit both siloxane (Si–O–Si) and silanol (SiOH) groups, whose ratio modules their hydrophilic/hydrophobic behaviour. Silica is a material that can be modelled adopting both periodic and cluster approaches.

Signorile et al. [177] studied the interaction of formamide (FA) on a well-defined amorphous silica model, the results of which were compared with IR measurements. The amorphous silica surface model was derived from a periodic 2D slab [178] with a moderate hydroxylation degree similar to the experimental sample (≈ 1.5 OH nm^−2^) represented by two independent surface silanol (SiOH) groups per unit cell. Calculations were performed at the PBE-D2/GTO level of theory. Authors docked FA on the silica surface maximising H-bond interactions considering the adsorption of up to two FA molecules, hence mimicking low and high coverage regimes. For the adsorption of two FA molecules, two possible cases were accounted for: adsorption of a double monomer (i.e., two non-interacting FA molecules) and adsorption of the FA dimer. For low coverage, two relevant adducts were identified: adsorption parallel to the surface and adsorption perpendicular to the surface. Obviously, dispersion interactions were found to be more relevant in the parallel adsorption mode. Using the last release of the CRYSTAL program [179,180] which is able to coherently treat molecules, polymers, slabs, and crystals at the DFT level using a Gaussian-type basis set, the authors also computed the vibrational frequencies and thermochemical corrections, which allowed obtaining free energy values of the adsorption process (ΔG). For FA-parallel and FA-perpendicular adducts, computed ΔG values were −5.1 and −4.6 kcal mol^−1^, respectively. From these values, the Boltzmann populations were calculated (0.67 and 0.30, respectively), indicating that both complexes can coexist. For high coverage, different adducts were explored and the final results indicated that the most stable adduct adopted a double monomer adsorption, in which each FA interacted independently with one surface SiOH group. The second most stable adduct was the adsorption of the FA dimer, simultaneously interacting with the two SiOH groups. Calculated ΔG values were −4.3 and −2.9 kcal mol^−1^, giving rise to a Boltzmann population of 0.90 and 0.08, respectively. By comparing the simulated IR spectra with those recorded experimentally, authors found that, in the low coverage regime, the experimental bimodal shape of the ν(C=O) band was due to the coexistence of the two identified adducts. In high coverage regime, the comparison between theory and experiment was less satisfactory, since the calculated ν(C=O) value was too low compared with experiment (about 20 cm^−1^), although the band profiles were reasonably coincident.

Tsendta et al. [181] studied the adsorption of a set of aromatic nitrogen-containing compounds, featured to be highly energetic chemicals such as TNT, with a cluster model of the (100) α-quartz surface using different DFT methods and MP2. Although the context of the work was related to environmental problems, main conclusions of the work can be extrapolated to nucleobases, the interaction of which with silica surfaces is, surprisingly, still missing. All compounds were physisorbed on the silica surface model through the formation of multiple H-bonds between the heteroatoms of the aromatic compounds and surface silanol groups. Parallel orientation of the compounds interacting with the silica surface was found to be more favourable than perpendicularly ones, due to the larger contribution of dispersion forces.

**Figure 10:**
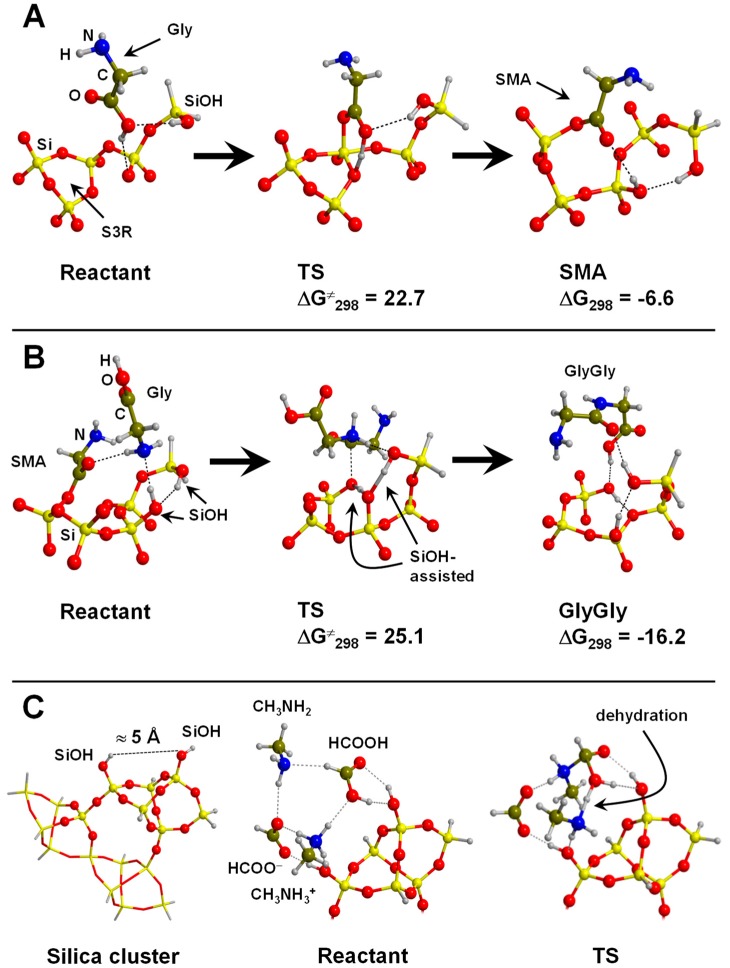
(**A**) Stationary points involved in the formation of the surface mixed anhydride (SMA) group from reaction of one glycine molecule (Gly) with a S3R ring. (**B**) Stationary points involved in the formation of the peptide glycylglycine (GlyGly) from reaction of SMA with a second Gly. Units of the relative free energies at 298 K are in kcal mol^−1^. (**A**,**B**) are adapted from [183]. (**C**) Optimized cluster model exhibiting the weakly interacting SiOH groups (left), the reactant structure for the formation of amide in which the canonical and ion pairs are shown (centre), and the optimized transition state for the dehydration step, which is initiated by a H-transfer from the CH_3_NH_3_^+^. Adapted from [58].

As far as reactivity on silica is concerned, the most recent works are related to the peptide bond formation. In an experimental seminal work of Basiuk et al. [182], activation of amino acids by reaction with silica surfaces forming a surface mixed anhydride (SMA, Si_surf_–O–C(=O)–) group was proposed. A theoretical attempt to explain the formation of SMA due to reaction of Gly with a surface SiOH group (modelled with a cluster structure containing an isolated SiOH) was carried out by us [174]. Results indicated that the process was endergonic by 6 kcal mol^−1^ at 298 K with a relatively high free energy barrier (23.6 kcal mol^−1^), thus ruling out this chemical channel. In a more recent work [183], SMA was proposed to be a product of the reaction between Gly and strained ring defects (namely, (SiO)_2_ and (SiO)_3_, referred to as S2R and S3R, respectively) present at the silica surface. This kind of defects can be formed by condensation between two silanol groups (i.e., Si–OH + Si–OH → Si-O–Si + H_2_O) when silica samples are treated at high temperature. Theoretical calculations demonstrated that these strained ring defects are indeed reactive toward COOH–containing molecules [184], including Gly [183]. The reaction involved the opening of the ring and formation of SMA and a SiOH group (see Figure 10A, which represents the calculated reaction for the S3R case). For the Gly case, the reaction was shown to be thermodynamically and kinetically more favourable with S2R than with S3R (free energy barriers of 4.6 and 22.7 kcal mol^−1^ and reaction free energies of −22.7 and −6.6 kcal mol^−1^) due to the larger strain of S2R. However, formation of S2R rings implies thermal treatments of about 900 K while S3R are expected to be formed at much lower temperatures. Accordingly, in a real silica sample, amounts of S2R are actually small while S3R rings are more abundant. The subsequent step studied by the authors was the peptide bond formation by reaction of SMA with an incoming Gly. This reaction can be assisted by SiOH groups (i.e., playing the same role of water in water-assisted reactions, see Figure 10B), reducing the energy barriers to 25.1 kcal mol^−1^. The role of silica was twofold: (1) immobilization of one glycine molecule via SMA formation, and (2) a decrease of the energy barrier of the peptide bond formation as the reactions occurred through a silanol-assisted proton-relay mechanism.

In a very recent work [58] experiments and simulations were combined to study the amide bond formation between amines and carboxylic acids in the presence of silica surfaces. IR measurements indicated that the reaction only took place if silica surface exhibited specific weakly interacting SiOH pairs, the presence of which were identified by thermally treating at 723 K the silica sample. Comparison of the experimental silanol ν(OH) vibrational frequencies with the calculated ones allowed establishing that the weakly interacting SiOH pairs should stay at ca. 5 Å apart (see Figure 10C, silica cluster model). Calculations also characterized the amide bond formation mechanism, using CH_3_NH_2_ and HCOOH as reactants. Results indicated that the weakly interacting SiOH are key specific sites to adsorb the reactants both in their canonical and ionic pairs; that is, CH_3_NH_2_/HCOOH and CH_3_NH_3_^+^/HCOO^−^, respectively (see Figure 10C, reactant structure). According to the computed mechanism, this specific coexistence was found to be fundamental for the occurrence of the reaction, in which the canonical pair was the reacting one, while the ionic one acted as a catalyst in the dehydration step (see Figure 10C, TS structure): the computed free energy barrier at 323 K (the T at which the experiments were performed) was 18.5 kcal mol^−1^. As a final outcome, authors demonstrated from an atomistic point of view that surfaces of naturally-occurring forms of silica (e.g., α-quartz) can also exhibit these specific weakly interacting SiOH pairs, thus suggesting that the explored mechanism is more general and could have been operative at the surface of crystalline silica phases present in early Earth under prebiotic conditions.

Silicates form a class of inorganic materials with a large diversity in chemical composition and structural properties. They consist of the [SiO_4_]^4−^ building block, in which the negative net electrical charge is compensated with metal cations such as Mg^2+^, Na^+^ or Al^3+^. They can also be understood as the combination of SiO_2_ with metal oxides. For instance, forsterite (Mg_2_SiO_4_) can be considered as a mixture of silica (SiO_2_) with magnesium oxide (MgO), i.e., SiO_2_ + 2MgO → Mg_2_SiO_4_. Silicates without the presence of OH groups are called anhydrous silicates, while those containing OH groups are referred to as phyllosilicates (or hydrous silicates) because they arise from the incorporation of water into anhydrous silicates. One of the most interesting groups of silicates in the prebiotic context are olivines, with general formula Mg_2x_Fe_(2−2x)_SiO_4_ (x = 0–1). In addition to belonging to the Earth crust, they are primary materials in interstellar and interplanetary dust particles, meteorites and comets [21,30]. Because of this relevance, as well as its structural simplicity, forsterite (the Mg-pure olivine, Mg_2_SiO_4_) has been used as a silicate of reference to study their interaction with organic compounds. It is important to mention that the outermost ions at forsterite surfaces are undercoordinated Mg atoms (capable of interacting favourably with electron donor atoms) and O atoms (capable of establishing H-bond interactions) [185,186,187].

**Figure 11:**
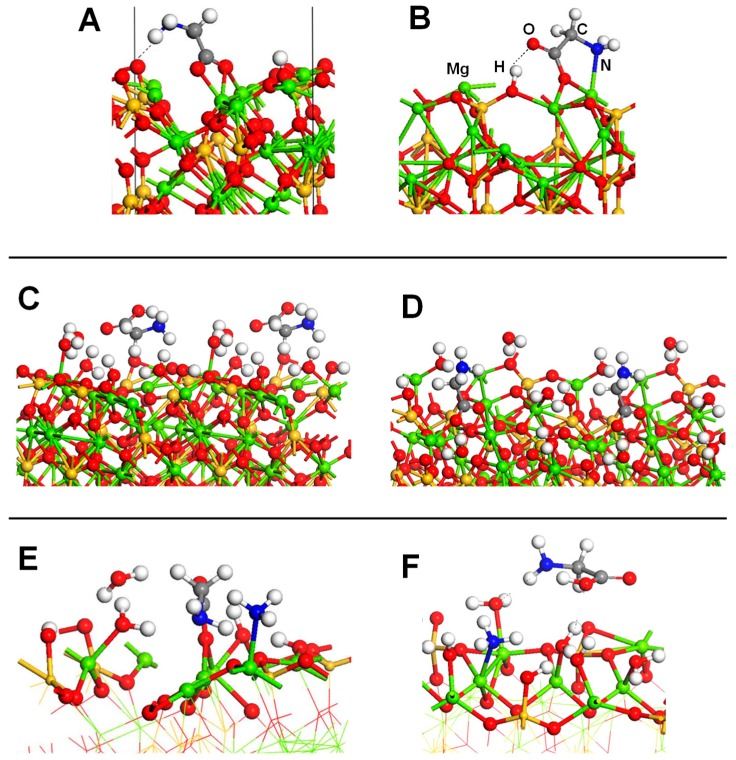
Different adsorption modes of glycine on the (100) forsterite (Mg_2_SiO_4_) surface in: dry conditions ((**A**,**B**), adapted from [188]), in the presence of a pure H_2_O layer ((**C**,**D**), adapted from [189]), and in the presence of a H_2_O/NH_3_ mixture ((**E**,**F**), adapted from [190]).

Escamilla–Roa and co-workers performed a set of studies focused on the interaction of Gly with the non-polar and polar forsterite (100) surfaces, both in dry conditions [188] and in the presence of thin layers of H_2_O [189] and H_2_O/NH_3_ [190]. Forsterite surfaces were modelled adopting a periodic approach at the PBE/PWs theory level. In dry conditions, the most stable adsorption adducts of Gly on both non-polar and polar surfaces presented the amino acid in a deprotonated state (see Figure 11A,B). On the non-polar surface Gly adsorbed by adopting a (O,O) mode, while on the polar surface this occurred through a (N,O) mode, with calculated adsorption energies of −66.7 and −96.1 kcal mol^−1^, respectively. In the presence of a thin H_2_O layer (modelled by the insertion of 5 H_2_O molecules) [189], authors studied two extreme cases: (1) Gly adsorption on the mineral surface covered by the H_2_O layer, and (2) simultaneous adsorption of Gly and the H_2_O layer (see Figure 11C,D, respectively). Results indicated that this second scenario is more favourable because the interaction of Gly on the H_2_O layer was weaker (only H-bonds were possible, interaction energy of −18.5 kcal mol^−1^) compared with the direct interaction of Gly with the silicate surface and the surrounding water molecules (both dative covalent and H-bond interactions were established, interaction energy of −157.9 kcal mol^−1^). In this latter case, Gly was initially adsorbed in canonical form but it transformed into the zwitterionic one upon geometry optimization. Authors, moreover, studied the transition from scenario (1) to scenario (2) and results indicated a progressive stabilization of the system towards scenario (2). In another work [190], layers of H_2_O/NH_3_ were modelled. Results indicated that interaction of Gly was more favourable when it was adsorbed simultaneously with the H_2_O/NH_3_ layers than on top of the H_2_O/NH_3_ layer covering the surface (calculated adsorption energies of −197.7 and −19.7 kcal mol^−1^, respectively, see Figure 11E,F). The presence of NH_3_ enhanced the stability of Gly on the surface compared with the pure H_2_O case.

Rimola et al. [191] studied the interaction of the (101) forsterite surface with 14 different organic compounds, which are representatives of the class of soluble organic compounds found in carbonaceous meteorites. Calculations were carried out adopting a periodic approach at the B3LYP-D2/GTOs level of theory. For all these compounds, different adsorption configurations were explored, in all cases (with the exception of aliphatic and aromatic compounds, the adsorption of which being essentially dictated by dispersion forces) maximising the dative interactions and the H-bonds. From the calculated adsorption energies, an intrinsic affinity ladder of the organic compounds with the forsterite surface was obtained, i.e., (from more to less favourable): phosphonic and sulfonic acids > carboxylic-containing molecules (glycine, and molecules containing the functional groups OHCOOH and –COOH) > amides > nitrogen heterocycles and amines (purines, pyrimidines and molecules containing the functional group –NH_2_) > carbonyl- and alcohol-containing molecules (glycolaldehyde, and molecules containing the functional groups –CHO and –OH) > aromatic and aliphatic hydrocarbons (C_6_H_6_ and C_4_H_10_). Interestingly, the two groups of molecules presenting the larger adsorption energies are both acidic compounds, in which the proton was transferred to the surface.

Fornaro et al. [192] studied the binding of nucleic acid components (i.e., nucleobases, nucleoside and nucleotides) on brucite (Mg(OH)_2_) both experimentally and using quantum chemical calculations. Brucite is a resulting product of the hydration of (Mg,Fe)_2_SiO_4_ olivines, the other products being serpentine (Mg,Fe)_3_Si_2_O_5_(OH)_4_ and magnetite Fe_3_O_4_. Simulations were carried out on the (110) brucite surface adopting a periodic approach at the B3LYP-D/GTO level. The (110) brucite slab model presented undercoordinated surface Mg atoms, which were saturated by H_2_O molecules. Results indicated that nucleotides adsorbed more favourably than nucleoside and nucleobases because in the former cases, interactions of the phosphate group with surface Mg cations were found to be strong enough to displace the saturating H_2_O molecules, while this was not the case for nucleosides and nucleobases.

**Figure 12:**
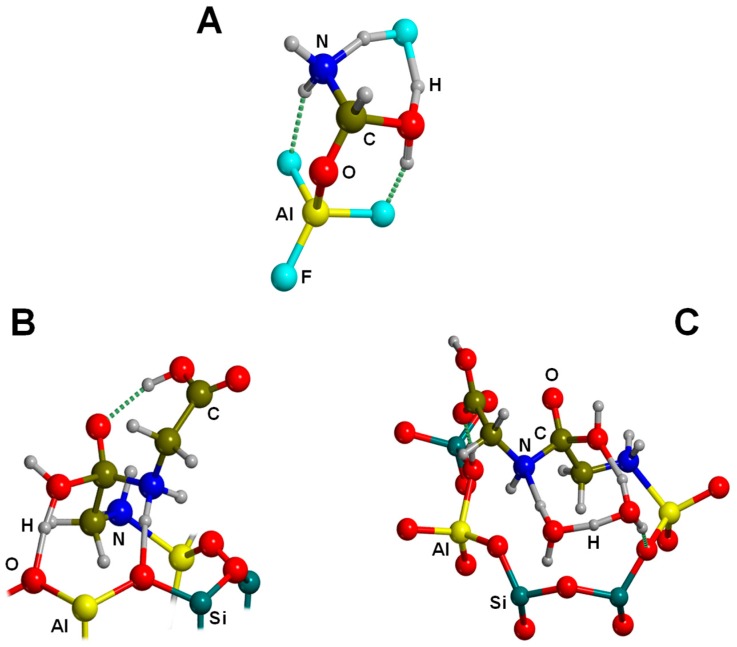
Transition state structures localized for: (**A**) the amide bond formation between HCOOH and NH_3_ under the synergy of AlF_3_ (Lewis acidic site) and HF (Brønsted acidic site), adapted from [173]; (**B**) the peptide bond formation between two glycine molecules on an acidic feldspar-derivative surface model, adapted from [175]; and (**C**): hydrolysis of the peptide bond on an acidic feldspar-derivative surface model assisted by three H_2_O molecules, adapted from [176].

Finally, several theoretical works addressed the peptide bond formation reaction catalysed by acidic aluminosilicates. Rimola et al. [173] showed that the interplay between Lewis and Brønsted acidic sites, eventually present at the mineral surfaces, may reduce the energy barrier for the amide (peptide) bond formation. In that work, the model reaction of NH_3_ + HCOOH → NH_2_CHO was simulated in the presence of a minimal cluster of AlF_3_ and HF, representing Lewis and Brønsted acidic sites in aluminosilicates, at B3LYP and CCSD(T) level of theory. Results indicated that when HCOOH was firmly attached to the Al atom and HF assisted the H-transfer (see Figure 12A) the free energy barrier at 298 K was dramatically reduced with respect to the uncatalyzed gas-phase reaction (i.e., 10.7 and 52.5 kcal mol^−1^). Authors found that such a decrease was the result of: (1) the interaction of the HCOOH with the Lewis site, enhancing the electrophilic character of the C atom, and (2) the proton-transfer assistance of HF, reducing the geometrical strain of the transition state. Obviously, the electronic effects exerted by the Lewis and Brønsted site models were exceedingly enhanced due to the size of the models and the presence of F dangling bonds, overestimating the electron withdrawing effects. Because of that, the same authors exploited the Lewis–Brønsted synergy using acidic feldspar surfaces as a test case for a more realistic mineral [175]. The surface model exhibited a coordinatively unsaturated Al atom as well as a H^+^ caused by an isomorphic substitution of [SiO_4_] by [AlO_4_]. The reaction to study was the condensation between two Gly molecules, in which one interacted with the two sites; i.e., the Al Lewis site through the N atom and the H^+^ surface site through the OH group. The reaction of this adduct with an incoming Gly molecule to form the dipeptide was found to take place through the transition state shown in Figure 12B. There, the N atom of the incoming Gly coupled to the C atom of the COOH group of the adsorbed Gly. Simultaneously, the surface H^+^ jumped to the OH group of the adsorbed glycine to yield the dehydration while one H atom of the attacking NH_2_ group jumped to the surface to recover the H^+^ site. This synchronism enabled the transition state to exhibit a non-strained eighth-membered ring. The calculated free energy barrier at 298 K was found to be 11.8 kcal mol^−1^, so that, at least for this specific feldspar surface model, the interplay between the two surface sites induced a significantly decrease of the free energy barrier. Finally, the hydrolysis of the dipeptide on the same surface model was also investigated [176]. The peptide hydrolysis was simulated considering that 3 H_2_O water molecules participated adopting a proton-transfer mechanism (see Figure 12C). Results showed that the reaction was endergonic by about 13 kcal mol^−1^ with a free energy barrier of 42 kcal mol^−1^, indicating that adsorption on the acidic feldspar surface protects the dipeptide from hydrolysis. A similar mechanism was simulated by Phuakkong et al. [193] for the peptide bond formation in a porous of the acidic Faujasite zeolite. Moreover, the reaction free energy was calculated to be exoergic by −9 kcal mol^−1^, thus indicating that the surface also favoured the thermodynamics of the reaction, probably due to the increased dispersion interactions between the peptide and the surface.

The following Table 5 and Table 6 summarize the results described so far. Reference is made to the internal tables and figures when present, otherwise the reference number is provided. To save space, we have adopted short notation. For instance, “Na^+^-free side: Montmorillonite/A; C; G; T; U” indicates that Na^+^-montmorillonite has been studied in interaction from its free side with adenine, cytosine, thymine, and uracil separately.

## 5. Conclusions and Perspectives

In the present work, the most recent in silico studies involved in prebiotic chemistry of the early Earth assisted by mineral surfaces have been reviewed. Four classes of minerals have been considered: clays, irons sulphides, titanium dioxide, and silica/silicates.

In relation to clays, most of the works mainly addressed the interaction of formamide and nucleobases. In contrast, for the other minerals, the biomolecules of reference were amino acids. Moreover, condensation of amino acids to form peptides is a key reaction also addressed in the presence of this class of minerals, in particular on FeS_2_, TiO_2_ anatase, silica, and aluminosilicate surfaces. The structures and interaction energies of the most representative adducts have been summarized. The driving forces for the adsorption of the organic compounds depend on the type of the mineral, but, as a trend, we can summarize they are: (1) H-bond interactions, between H-donor groups of the adsorbate and O (or S) atoms of the surfaces; (2) dative covalent bonds, between lone pair electrons of adsorbate atoms and coordinatively unsaturated surface metal cations; (3) dispersive interactions, between aliphatic/aromatic groups and non-polar regions of the surfaces; and (4) electrostatic interactions, in the case of metal-containing clays with isomorphic substitutions. In some works, water solvation has been considered and described with AIMD simulations to rigorously account for the dynamic effects ubiquitously present in liquid water. Interestingly, for the particular case of iron sulphides, ab initio metadynamics simulations were carried out to accurately account for the extreme conditions of the black smokers (i.e., hot-pressurized water), which were shown to be crucial for the formation of peptides.

Although all the reviewed (and other) works have helped to make breakthroughs in our understanding in the mineral-induced prebiotic chemical evolution, several research lines need to be investigated. As far as clays are concerned, interaction of formamide and nucleobases is almost covered. However, no chemical reactions have been simulated so far. For instance, the N-glycosidic bond formation (linking ribose with a nucleobase, only studied in the gas phase [194]) or the phosphoester bond formation (linking ribose with a phosphate group, never studied) in the interlayer region of clays have not been studied yet, which are fundamental reactions to assess any possible role of clays in the formation of nucleotides and their subsequent polymerization. Such polymerizations have been successfully reported experimentally [59]; accordingly, providing an atomistic perspective of these reactions will be of high relevance. In the same line, reactivity of formamide in presence of clays (and other minerals) will be of great relevance, since many experiments (e.g., [39]) provide clear evidence on the potentiality of formamide as a key molecule that gives rise a wide of reactions and compounds of prebiotic interest in the presence of different minerals. The theoretical works concerning formamide reactivity do not include the effect of the mineral surfaces [195,196]. A key point to investigate is whether formamide is decomposed first into HCN followed by its intrinsic reactivity or, on the contrary, reactions involved formamide as it is. Also for clays, information on the interaction of their surfaces with amino acids is very scarce. A systematic study to understand the influence of the side chains of the amino acids interaction with different interlayer surfaces (e.g., octahedral vs tetrahedral, influence of metal cations, or interaction with hydroxylated broken edges), including solvation effects, will provide general trends of great value, as done for e.g., silica [197] and anatase [169] surfaces. Possible catalytic effects of clays in the polymerization of peptides should also be explored, in view of the successful experiments [59].

For iron–sulphur mineral cases, as reviewed here, a lot of work has already been done, particularly in the interaction of glycine and its polymerization into peptides. However, these works were essentially in the presence of FeS_2_, in which Fe is in its oxidized state. One of the key points of the iron-sulphur world theory is the powerful redox properties inferred by the iron sulphide minerals. Accordingly, computational investigations considering FeS (with strong reducing properties) will be of high value. In the same line, possible redox effects inferred by Fe^2+^ in minerals (e.g., olivines) should be investigated, since they can be of relevance as already identified in problems of astrochemical interest [198]. Along the same line, it would also be important to investigate if the exergonicity exhibited by the FeS + H_2_S → FeS_2_ + H_2_ reaction can be used in subsequent reactions. Additionally, simulation of chemical reactive steps involved in surface metabolic cycles (e.g., synthesis of pyruvate or citrate, which are experimentally tested [42,43]) will serve to provide a robust evidence on the plausibility of the iron–sulphur world theory and an understanding of the role of the iron sulphides in the reactions. This, in turn, would open up an interesting research line practically unexplored in prebiotic chemistry investigations: the possible role of minerals as protoenzymes. Could minerals covered by short prebiotic building blocks have exhibited “enzymatic” functionalities that activated and triggered fundamental catalytic cycles relevant for autotrophic chemical evolution and complexification? Clues to answer this question can certainly be provided by quantum chemical simulations.

At variance with amino acids, the interaction of nucleobases with TiO_2_ surfaces, silica, and silicates is practically missing. Understanding the adsorption of these biomolecules with these mineral surfaces is the first step to subsequently studying their reactivity, e.g., N-glycosidic and phsophoester bond formation (as mentioned above). Similar comments can be done for the interaction and reactivity of formamide with these mineral surfaces. While solvation of TiO_2_ and silica is an important aspect that is well-studied and reported [82,199], this is not the case for silicate-based materials. Accordingly, AIMD simulations on the H_2_O-silicate and organic–silicate interfaces will be very useful for checking the influence of water solvent in the biomolecule–mineral interactions.

All these mentioned aspects are few points that we think need to be covered in the forthcoming years. They will be milestones to improve our current know-how of the prebiotic chemistry assisted by mineral surfaces, which in some cases can be a source of inspiration to derive and refine new catalysts useful to our society.

## Figures and Tables

**Table 1:** Report of calculated basis set superposition error (BSSE)-corrected adsorption energies (ΔE^C^_ads_, in kcal mol^−1^) for formamide interacting with octahedral and tetrahedral dickite and kaolinite mineral fragments.

Surface	Sheet	Level of Theory	ΔE^C^_ads_	Ref.
Dickite	Octahedral–adsorption	B3LYP/3-21G(d)	−14.5	[117]
	Octahedral–intercalation	B3LYP/3-21G(d)	−20.2	[117]
Kaolinite	Octahedral	M05-2X/6-31G(d)	−14.8	[119,120]
	Tetrahedral	M05-2X/6-31G(d)	−13.7	[119,120]
	Octahedral–water	M05-2X/6-31G(d)	−9.2	[119]
	Tetrahedral–water	M05-2X/6-31G(d)	−5.9	[119]
Na^+^-kaolinite	Octahedral	M05-2X/6-31G(d)	−108.2	[119]
	Tetrahedral	M05-2X/6-31G(d)	−20.3	[119]
	Octahedral–water	M05-2X/6-31G(d)	−21.7	[119]
	Tetrahedral–water	M05-2X/6-31G(d)	−17.8	[119]

**Table 2:** Report of calculated BSSE-corrected adsorption energies (ΔE^C^_ads_, in kcal mol^−1^) for uracil and thymine interacting with octahedral and tetrahedral dickite [122] and Na^+^-kaolinite [123] mineral fragments.

Surface	Sheet	Molecule	Level of Theory	ΔE^C^_ads_
Dickite	Octahedral	Uracil	B3LYP/6-31G(d)	−30.3
Dickite	Tetrahedral	Uracil	B3LYP/6-31G(d)	−3.6
Dickite	Octahedral	Thymine	B3LYP/6-31G(d)	−21.1
Dickite	Tetrahedral	Thymine	B3LYP/6-31G(d)	−1.4
Dickite	Octahedral–water	Uracil	B3LYP/6-31G(d)	−47.8
Dickite	Tetrahedral–water	Uracil	B3LYP/6-31G(d)	−8.6
Dickite	Octahedral–water	Thymine	B3LYP/6-31G(d)	-45.7
Dickite	Tetrahedral–water	Thymine	B3LYP/6-31G(d)	−8.2
Na^+^-kaolinite	Octahedral	Uracil	M05-2X/6-31G(d)^a^	−46.1
Na^+^-kaolinite	Tetrahedral	Uracil	M05-2X/6-31G(d)^a^	−31.0
Na^+^-kaolinite	Octahedral	Thymine	M05-2X/6-31G(d)^a^	−44.4
Na^+^-kaolinite	Tetrahedral	Thymine	M05-2X/6-31G(d)^a^	−29.9
Na^+^-kaolinite	Octahedral–water	Uracil	M05-2X/6-31G(d)^a^	−43.7
Na^+^-kaolinite	Tetrahedral–water	Uracil	M05-2X/6-31G(d)^a^	−28.5
Na^+^-kaolinite	Octahedral–water	Thymine	M05-2X/6-31G(d)^a^	−43.4
Na^+^-kaolinite	Tetrahedral–water	Thymine	M05-2X/6-31G(d)^a^	−27.0

**Table 3:** Report of calculated adsorption energies (in kcal mol^−1^) for the interaction of nucleobases with Na^+^-montmorollinite [124] and H^+^-montmorollinite [126]. Perpendicular and parallel adsorptions with respect to the external surface are indicated as ┴ and ║, respectively.

External Surface	Nucleobase	Adsorption Type	ΔE_ads_
Na^+^-free side	Adenine	┴	−3.7
		║	−10.8
	Cytosine	┴	−6.6
		║	−9.1
	Guanine	┴	−11.3
		║	−10.9
	Thymine	┴	−7.3
		║	−10.7
	Uracil	┴	−5.7
		║	−8.5
Na^+^-containing side	Adenine	Cation–π/ring	−11.6
		Cation–π/displaced	−17.0
		Cation–heteroatom	−20.2
	Cytosine	Cation–π/ring	−10.2
		Cation–π/displaced	−26.6
		Cation–heteroatom	−27.0
	Guanine	Cation–π/ring	−13.1
		Cation–π/displaced	−26.1
		Cation-heteroatom	−27.6
	Thymine	Cation–π/ring	−7.6
		Cation–π/displaced	−21.7
		Cation–heteroatom	−19.1
	Uracil	Cation–π/ring	−5.7
		Cation–π/displaced	−21.2
		Cation–heteroatom	−18.8
H^+^-montmorillonite	Adenine	║ on tetrahedral substituted	−37.7
		║ on octahedral substituted	−49.1
		┴ on tetrahedral substituted	−39.0
		┴ on octahedral substituted	−49.4
	Guanine	║ on tetrahedral substituted	−39.8
		║ on octahedral substituted	−48.4
		┴ on tetrahedral substituted	−40.7
		┴ on octahedral substituted	−50.0
	Cytosine	║ on tetrahedral substituted	−42.4
		║ on octahedral substituted	−44.0
		┴ on tetrahedral substituted	−32.3
		┴ on octahedral substituted	−41.8

**Table 4:** Report of calculated adsorption energies (ΔE_ads_, in kcal mol^−1^) for the interaction of amino acids with TiO_2_ surfaces. The different amino acid states have been considered: deprotonated, zwitterionic, and canonical. GTO: Gaussian-type orbital.

Surface	Amino Acid	Amino Acid State	Method	ΔE_ads_	Ref.
Rutile (110)	Glycine	Deprotonated	PW91/PWs	−48.5	[160]
	Glycine	Zwitterionic	PW91/PWs	−47.1	[160]
	Glycine	Deprotonated	PBE/PWs	−31.3	[162]
	Glycine	Zwitterionic	PBE/PWs	−29.2	[162]
	Proline	Deprotonated	PBE/PWs	−30.6	[162]
	Proline	Zwitterionic	PBE/PWs	−26.1	[162]
	Cysteine	Deprotonated	PBE/PWs	−33.9	[166]
	Cysteine	Zwitterionic	PBE/PWs	−31.1	[166]
Anatase (101)	Glycine	Deprotonated	PBE0/GTO	−25.6	[168]
	Glycine	Zwitterionic	PBE0/GTO	−17.6	[168]
	Glycine	Canonical	PBE0/GTO	−26.7	[168]
	Glycine	Deprotonated	PBE/PWs	−25.9	[169]
	Glycine	Zwitterionic	PBE/PWs	−24.0	[169]
	Glycine	Canonical	PBE/PWs	−23.4	[169]

**Table 5:** Summary of results for clays and iron sulphide studies. Quantum mechanical adopted methods (QM method), structural approach (SA), computed quantities (Obs.) and internal references (Reference) of the considered cases. Captions: S: structure; AE: adsorption energy; RE: reaction energy; FRE: free energy of reaction; V: vibrational spectrum; FA: formamide; A: adenine; C: cytosine; G: guanine; T: thymine; U: uracil; UD: uridine; UPM: uridine-5′-monophosphate; AMP: adenosine-5′-monophosphate. PBC: periodic boundary conditions. CLU: cluster.

CLAYS (Section 4.1)	QM Method	SA	Obs.	Reference
Dickite/FA (adsorption/intercalation)	B3LYP/3-21G(d)	CLU	S, E	Table 1
Dickite/U; Dickite/U-H_2_O	B3LYP/6-31G(d)	CLU	S, E	Table 2, Figure 4A,B
Dickite/T; Dickite/T-H_2_O	B3LYP/6-31G(d)	CLU	S, E	Table 2
Na^+^-Kaolinite/FA	MO5-2X/6-31G(d)	CLU	S, E	Table 1
Na^+^-Kaolinite/U; Na^+^-Kaolinite/U-H_2_O	MO5-2X/6-31G(d)	CLU	S, E	Table 2, Figure 4C–F
Kaolinite/FA; Kaolinite/FA/H_2_O	M05-2X/6-31G(d)	CLU	S, E, V	Table 1, Figure 3
Kaolinite/N-methylacetamide	BLYP/SVP + sp	CLU	S, E, V	Ref. [121]
Na^+^-free side: Montmorillonite/A; C; G; T; U Na^+^-containing side: Montmorillonite/A; C; G: T; U Na^+^-Montmorillonite/C-H_2_O H^+^-Montmorillonite/A; G; C	PBE-D2/PWs	PBC	S, E	Table 3, Figure 5
K^+^-Montmorillonite/GLY GLY^+^-Montmorillonite/GLY	PBE-D2/PWs-Num	PBC	S, E	Ref. [128,129]
**IRON SULPHIDES (Section 4.2)**	**QM Method**	**SA**	**Obs.**	**Reference**
Violarite (Fe,Ni)S + CH_3_SH + CO + H_2_O→CH_3_COOH + H_2_S	B3LYP/TZVP	CLU	S, RE	Figure 2E
FeS_2_-defective (100)+NO_3_^−^→NH_3_	AIMD/PBE-D2/PWs + GTO	PBC	S, FRE	Figure 6
FeS_2_ (100)/GLY FeS_2_-defective (100)/GLY	AIMD/PBE/PWs	PBC	S, FRE	Figure 7A,B
FeS_2_-defective (100) + GLY + COS→(GLY)_n_ + H_2_S	AIMD/PBE/PWs	PBC	S, FRE	Figure 7C
FeS+nGLY→(GLY)_n_	AIMD/PBE/PWs	PBC	S, FRE	Ref. [152]

**Table 6:** Summary of results for titanium dioxide and silica and silicates studies. Quantum mechanical adopted methods (QM method), structural approach (SA), computed quantities (Obs.) and internal references (Reference) of the considered cases. Captions: S: structure; AE: adsorption energy; RE: reaction energy; FRE: free energy of reaction; V: vibrational spectrum; FA: formamide; A: adenine; C: cytosine; G: guanine; T: thymine; U: uracil; UD: uridine; UPM: uridine-5′-monophosphate; AMP: adenosine-5′-monophosphate. PBC: periodic boundary conditions. CLU: cluster.

TITANIUM DIOXIDE (Section 4.3)	QM Method	SA	Obs.	Reference
Rutile (110)/GLY	PW91/PWs	PBC	S, E	Table 4
Rutile (110)/GLY/CYS/PRO	PBE/PWs	PBC	S, E	Table 4
Rutile(OH) (100)-(110)-H_2_O/GLY/MET/SER/CYS	AIMD/PWs	PBC	S, E	Ref. [161]
Anatase (101)/GLY	PBE/PWs	PBC	S, E	Table 4, Figure 8
Anatase (101)/LEU/MET/PHE/SER/CYS/GLU/GLN/LYS/HIS/ARG	PBE/PWs	PBC	S, E	Ref. [169]
Anatase (101)/GLY	PBE0/GTO, PBE/PWs	PBC	S, E	Table 4, Figure 8
Anatase (101)/2GLY→GLY_2_	PBE/PWs	PBC	S, E	Figure 9
Anatase-O-defective (001)/2H_2_CO→HOCH_2_CHO	PBE/PW	PBC	S, E	Ref. [171]
**SILICA & SILICATES (Section 4.4)**	**QM Method**	**SA**	**Obs.**	**Reference**
a-SiO_2_/FA-(FA)_2_	PBE-D2/GTO	PBC	S, E, V	Ref. [177]
α-quartz (100)/aromatic N-containing molecules	DFT/MP2/GTO	CLU	S, E	Ref. [181]
a-SiO_2_(defective)/Gly→surface mixed anydride	B3LYP/GTO	CLU	S, RE, V	Figure 10A,B
a-SiO_2_/CH_3_NH_2_+HCOOH→GLY+H_2_O	B3LYP/GTO	CLU	S, RE, V	Figure 10C
Mg_2_SiO_4_(100)/GLY/H_2_O/NH_3_	PBE/PWs	PBC	S, E	Figure 11
Mg_2_SiO_4_(101)/14 organic compounds	B3LYP-D2/GTO	PBC	S, E	Ref. [191]
Mg(OH)_2_ (110)/U/UD/UPM/AMP	B3LYP-D2/GTO	PBC	S, E	Ref. [192]
AlF_3_/HF+NH_3_+HCOOH→GLY+H_2_O	B3LYP/GTO CCSD(T)/GTO	CLU	S, RE	Figure 12A
Feldspar (SiOHAl)+2GLY→GLY_2_+H_2_O	ONIOM(B3LYP//MNDO)	CLU	S, RE	Figure 12B
Feldspar (SiOHAl)-GLY-GLY+3H_2_O	ONIOM(B3LYP//MNDO)	CLU	S, RE	Figure 12C
Faujasite+2GLY→GLY_2_+H_2_O	M08-HX/GTO	CLU	S, RE	Ref. [193]
